# Quantitative Detection of Digoxin in Plasma Using Small‐Molecule Immunoassay in a Recyclable Gravity‐Driven Microfluidic Chip

**DOI:** 10.1002/advs.201802051

**Published:** 2019-01-27

**Authors:** Hailong Li, Jesper Vinther Sørensen, Kurt Vesterager Gothelf

**Affiliations:** ^1^ Center for DNA Nanotechnology Interdisciplinary Nanoscience Center, iNANO Aarhus University Gustav Wieds Vej 14 8000 Aarhus C Denmark; ^2^ Department of Chemistry Jilin University Changchun 130012 China; ^3^ Department of Chemistry Aarhus University Langelandsgade, 140 8000 Aarhus C Denmark

**Keywords:** beads, digoxin, gravity‐driven microfluidic chips, two signal‐mode immunoassays

## Abstract

Immunoassays are critical for clinical diagnostics and biomedical research. However, two major challenges remaining in conventional immunoassays are precise quantification and development of immunoassays for small‐molecule detection. Here, a two signal‐mode small‐molecule immunoassay containing an internal reference that provides high stability and reproducibility compared to conventional small‐molecule immunoassays is presented. A system is developed for quantitative monitoring of the digoxin concentration in plasma in the clinically relevant range (0.6–2.6 nm). Furthermore, the model system is integrated into a simple gravity‐driven microfluidic chip (G‐Chip) requiring only 10 µL plasma. The G‐Chip allows fast detection without any complex operation and can be recycled for at least 50 times. The assay, and the G‐Chip in particular, has the potential for further development of point‐of‐care (POC) diagnostics.

## 1 Introduction

Immunoassays have a wide range of applications in clinical diagnostics and molecular biology due to the unique specificity, sensitivity, and flexibility.^1^ Conventional immunoassays only rely on one‐signal readout channel for (semi)quantification. Moreover, quantitative and specific detection of small molecules remains a great challenge since the conventional sandwich immunoassay, which is capable of a 1000‐fold improvement in detection limit, is not compatible with small‐molecule targets.^2^ Quantification in particular can be important for small‐molecule drugs that have a narrow therapeutic range, because the assessment of the actual blood concentration in patients can be critical. Furthermore, detection of small molecules with applications in metabolic pathway optimization, metabolite concentration measurement and imaging, environmental toxin detection, and small molecule–triggered therapeutic response is also of interest.^3^ The most widely used method for measuring concentrations of small molecules is high‐performance liquid chromatography in combination with mass spectrometry (MS), UV, and/or fluorescence detection. These methods are very precise and reliable, but require specialized laboratories and personnel that are trained in the laborious and time‐consuming operation.^4^

Here, we provide a competitive heterogeneous immunoassay that exploits an internal reference to obtain higher precision, stability, and reliability in comparison with conventional one‐signal immunoassay. The two signal‐mode immunoassay is used to demonstrate the detection of the small‐molecule drug digoxin (Dig).^5^ Digoxin is a cardiac glycoside used to treat cardiac arrhythmia and congestive heart failure. It has been used for more than 200 years and is still one of the most widely prescribed heart failure drugs. However, strict control and monitoring of digoxin is required to minimize the risk of toxicity because of its narrow therapeutic range of 0.6–2.6 nm.^6^ Here, we demonstrate a detection range for digoxin of 0.2–6 nm. The assay is applied to detection of digoxin in plasma of human blood, and it is further integrated into a recyclable gravity‐driven microfluidic chip (G‐Chip).

## 2 Result and Discussion

### 2.1 Assay Design for Detection of Digoxin

**Figure 1:**
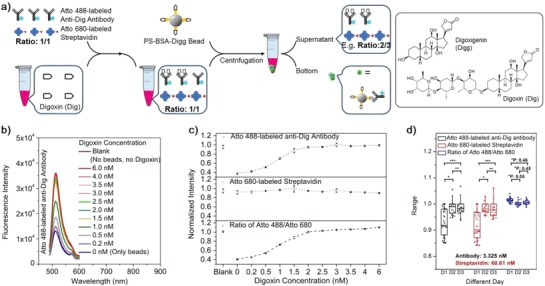
Illustration of the method and responses of the assay to digoxin at different concentrations. a) The principle of digoxin detection. b) Fluorescence emission spectra of Atto 488‐labeled anti‐Dig antibody (3.33 nm) from the supernatant after being incubated with different concentrations of digoxin, subsequent incubation with PS–BSA–Digg beads (10 µL), and followed by removal of the beads. c) Normalized fluorescence intensities of Atto 488 and Atto 680. The signal ratio of Atto 488/Atto 680 is calculated accordingly. d) Investigation of the advantages of the two signal‐mode strategy over single signal‐mode measurements by comparing day‐to‐day variation of the detection system. Box and whisker plots of the normalized fluorescence intensity and corresponding signal ratio. Horizontal lines are medians, boxes show the interquartile range (IQR), and error bars show the full range excluding outliers (crosses) defined as being more than ±1.5 IQR outside the box. Asterisks indicate statistically significant differences (*P* < 0.05) in day‐to‐day variance; *P* values are given where there are no significant differences (*P* > 0.05). Plotted values are mean values with standard deviations compared to the mean value (*N* = 3).

The detection mechanism of the competitive immunosorption assay is illustrated in **Figure** **1**a. First, the sample containing the analyte digoxin is preincubated with the fluorescent probes: Atto 488‐labeled anti‐Dig antibody and Atto 680‐labeled streptavidin. The antibody of the former is inhibited by digoxin, while the latter serves as reference. Polystyrene (PS) carboxylate beads (20 µm) are modified with digoxigenin (Digg), the aglycon moiety of digoxin, which binds to the anti‐Dig antibody with an affinity similar to that of digoxin. Digg is linked to the beads via covalent coupling to bovine serum albumin (BSA), which in turn is coupled to the particles.^7^, ^8^ The PS–BSA–Digg beads are added to the solution to bind excess Atto 488‐labeled anti‐Dig antibody, i.e., antibodies which have not been inhibited by digoxin, to facilitate removal by centrifugation. The fluorescent signal from the Atto 488‐labeled antibody remaining in solution, divided by the signal from the labeled streptavidin reference in the supernatant, is proportional to the digoxin concentration in the original sample. The ratio between the two fluorescent signals enables correction for environmental effects and thus improves sensitivity and is used to determine digoxin concentration.^9^, ^10^, ^11^, ^12^

The fluorescent signals of both Atto 488 and Atto 680 at various probe concentrations were measured repeatedly over the course of three days and signal ratios of Atto 488 to Atto 680 were calculated (see Section S1, Supporting Information, for detailed day‐to‐day, sample‐to‐sample, concentration‐to‐concentration analyses). Figure 1d shows the summarized distribution and variation from day‐to‐day comparison. The distribution range in the normalized intensity *d_I_* is given by the normalized maximum intensity (*I*_max_) minus the normalized minimum intensity (*I*_min_). From single signals recorded at the same day, the *d_I_* is 0.060–0.262 for Atto 488‐labeled anti‐Dig antibody and 0.063–0.250 for Atto 680‐labeled streptavidin. However, the distribution range for the signal ratio of Atto 488/Atto 680 with normalized intensity is between 0.014 and 0.097, which is much narrower. In addition, all the *P* values are calculated for day‐to‐day comparison and they are equal to or above 0.05 for the signal ratio comparison.

The method proved feasible for digoxin detection (Section S2, Supporting Information), and the optimal concentration of Atto 488‐labeled anti‐Dig antibody probe for detection of digoxin in the therapeutic range of 0.2–6 nm is found to be 3.3 nm. At this concentration, we investigate the optimal amount of PS–BSA–Digg beads. In the absence of digoxin, a series of experiments with variable amounts of beads from 0 to 12 µL (about 1.5% solid w/v) are prepared to identify the optimal ratio between beads and probe which gives in the lowest background signal in detection buffer (see Section S3, Supporting Information). When reaching 10–12 µL of the beads, the signal ratios are almost identical. The remaining background may arise from inactive dye‐labeled antibody. Based on the results, 10 µL PS–BSA–Digg beads are chosen as the optimal amount for further experiments under the probe concentration of ≈1.52 × 10^4^ µL beads nmol^−1^ probe antibody. The immunoadsorption is almost completed in 20–30 min (see kinetics investigation in Section S3, Supporting Information). Hereafter, we investigate detection of digoxin at different digoxin concentrations (Figure 1b). The samples are first preincubated with probes before PS–BSA–Digg beads are added to bind excess Atto 488‐labeled anti‐Dig antibody. From the signals in the supernatant, the fluorescence intensity from Atto 488‐labeled antibody gradually becomes stronger with the increment of digoxin concentration (Figure 1b) while the fluorescence intensity from Atto 680‐labeled streptavidin only shows a small variation (see Section S3, Supporting Information). The normalized fluorescence and the corresponding signal ratio are shown in Figure 1c. The signal ratio provides a dynamic and reliable response to different concentrations. The present method has a detection range of 0.2–6 nm with a practical detection limit of 0.2 nm (Section S3, Supporting Information), which covers the range of clinical monitoring of digoxin. It should be noted that the simulations associated with the optimization of the amount of beads, kinetics, and digoxin detection here are quite consistent with the experimental results (see Section S4, Supporting Information). Also, refer Table S3 in the Supporting Information for detailed comparison between the current method and last 5 year literatures as well as associated commercial kits.^13^, ^14^, ^15^, ^16^, ^17^

### 2.2 Specificity of Digoxin Detection

**Figure 2:**
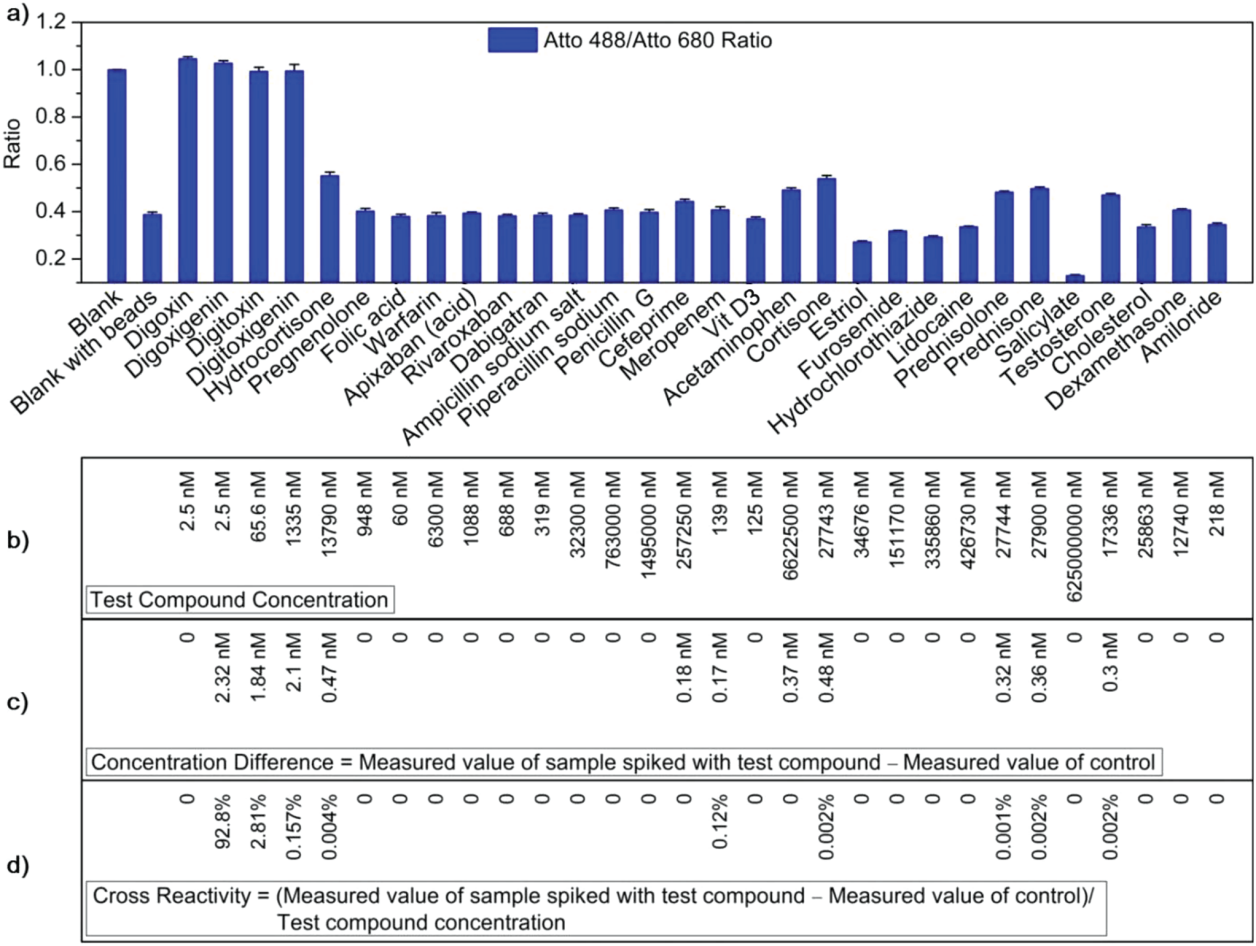
Investigation of specificity and assay precision. a) Atto 488/Atto 680 signal ratios after normalizing fluorescence emission intensity of Atto 488‐labeled anti‐Dig antibody (3.33 nm) and Atto 680‐labeled streptavidin (60.61 nm) from the detection system in response to different molecules of the same kind of drug or with analogous structure. b) The concentration of each investigated small molecules in the detection system. c) The calculated CD for each molecule. d) Calculated CR for each molecule. Plotted values are mean values with standard deviations compared to the mean value (*N* = 3).

The specificity of the detection method is further investigated by treating the system with a series of compounds. These compounds either have similar or related structure, or are pharmaceutically relevant. The responses of the system are processed from samples spiked with individual substances (**Figure** **2**a; see details in Section 5, Supporting Information).

The Concentration Difference (CD) is calculated for each molecule according to Equation (1), and the Cross Reactivity (CR, given by Equation (2)) is calculated by deriving a ratio between CD and the tested compound concentration. From the results shown in Figure 2c,d, it appears that the interference of all the selected compounds on the detection system is minimal.

(1) $$\begin{array}{l} \mathrm{CD}=\mathrm{Measured digoxin concentration of sample spiked with test}\\ \;\;\;\;\;\;\;\;\;\mathrm{compound}\left(C_{\mathrm{Test}}\right)-\mathrm{Digoxin concentration of control}\;\left(C_{\mathrm{Ctrol}}\right) \end{array}$$

(2) $$\mathrm{CR}\;=\frac{\mathrm{Concentration}\;\mathrm{difference}}{\mathrm{Test}\;\mathrm{compound}\;\mathrm{concentration}}$$

As expected, the response of the system to digoxin and digoxigenin is almost the same because they share main structure which is recognized by the labeled antibody. Although digitoxin and digitoxigenin give high fluorescence responses, due to their structural relation to digoxin, the corresponding CR is low at administered concentrations. All the results in Figure 2a–d indicate high specificity of the developed method for digoxin detection. The assay precision is further investigated by analyses on three different levels of pool control samples. **Table** **1** shows the number of test times, spiked concentration, mean values determined, standard deviation (SD), and coefficient of variation (CV) for each of these control samples. The method has an assay precision of <10% CV (with mean around 5%).

**Table 1:** Assay precision investigation at three different levels of concentration

Concentration	Number	Spiked [nm]	Mean [nm]	SD	CV
Low	10	0.6	0.563	0.02	3%
Middle	10	1.6	1.51	0.1	7%
High	10	2.6	2.42	0.13	6%

### 2.3 Recycling of the PS–BSA–Digg Beads

To study recycling of the PS–BSA–Digg beads, the collected beads used in the prior experiments are incubated with a commercial dissociation buffer that disrupts the binding between anti‐Dig and digoxin/digoxigenin (see Section S6, Supporting Information). The dissociation of anti‐Dig from the PS–BSA–Digg beads is very fast. After 1 min, the dissociation of the anti‐Dig antibody from the beads is almost complete and it takes only 10–15 min to reach the same background as fresh PS–BSA–Digg beads. To assess whether the PS–BSA–Digg beads conserve their activity after the dissociation procedure, the recycled beads were applied to the detection system again. In Figures S15b,c and S17b in the Supporting Information, it appears that the recycled beads treated with dissociation buffer from 15 s to 3.5 h are good as fresh beads. Longer dissociation times at 1–3 h do not destroy the modified beads or influence the activity. In the experiments shown in Figures S16 and S17c in the Supporting Information, the beads are recycled 15 times. Furthermore, all the following experiments were done by recycling the beads up to 50 times. The error bars from the signal ratios only increase slightly after extended recycling of the beads, because the recycling process involves many rounds of washing and centrifugation, which may cause some loss of beads in the process.

The performance of recycled beads in the detection of digoxin at different concentrations is further investigated (Section S7: Figures S18–S21, Supporting Information). Although the concentration regression curve is a little different from that with fresh beads (Figure S21a,e, Supporting Information), the difference is not significant and it might be ascribed to the following reasons: i) there is a slight change of the modified beads' surface because the linked BSA is somewhat denatured in the process of recycling; ii) there may be trace of chemical residues from the immobilization of digoxigenin in the suspension of fresh beads, but they are removed in the process of recycling.

### 2.4 Detection of Digoxin in Human Plasma

Detection of digoxin at different concentrations is performed with detection buffer containing 5% and 15% plasma, respectively (see Section S7, Supporting Information). From the comparison of the plotted concentration regression curves derived from detection in buffer, 5% plasma, and 15% plasma, it is evident that the influence of plasma on the detection is minimal (it should be noted that the beads are also recycled in these experiments). The responses of the system to 1.5 nm digoxin in 25% and 50% plasma are also investigated. A concentration of digoxin of 1.5 nm is chosen for spiking because it is at the most sensitive area in the detection range (almost the inflexion point of the fitting curve). It turns out that there is no significant difference in the response of the assay to samples spiked with 1.5 nm digoxin, in buffer or in 5% or 15% plasma (see Section S7, Supporting Information).

**Figure 3:**
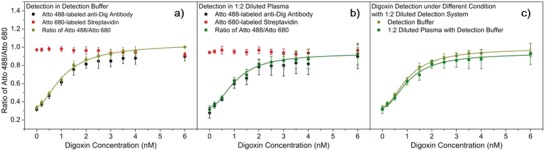
Digoxin detection in plasma diluted 1:2. Plots of normalized fluorescence intensity of Atto 488‐labeled anti‐Dig antibody (1.11 nm) and Atto 680‐labeled streptavidin (20.20 nm) (1:2 diluted probe system), and the corresponding signal ratio in digoxin detection under different conditions: a) detection buffer; b) diluted plasma (1:2) with detection buffer (about 60 µL of plasma is needed in each sample). c) Comparison of plotted concentration regression curves from (a) and (b). The concentrations given in all graphs are before dilution. We used recycled PS–BSA–Digg beads. Plotted values are mean values with standard deviations compared to the mean value (*N* = 3).

We further pursue the detection of digoxin in plasma from blood. The viscosity of pure plasma makes it difficult to separate the modified beads from the solution, and hence the assay works better with diluted plasma. Fortunately, also as another advantage of the suggested two‐signal strategy, the final signal ratio does not depend much on the dissolution (Figure S23 in Section S7, Supporting Information). There is no significant difference in the final signal ratio when it is diluted 1:2 and the variation extent is below 20% even when the detection system is diluted 1:9. Accordingly, Protocol 1a in the Supporting Information is set up to determine the digoxin concentration in plasma. The results for digoxin in buffer and 33% plasma are shown in **Figure** **3**. In this protocol, pure plasma and the probe concentrations for the detection system are diluted 1:2. The concentration regression curves obtained in buffer and in diluted plasma are almost identical.

### 2.5 Gravity‐Driven Microfluidic G‐Chip for Detection of Digoxin in Plasma

**Figure 4:**
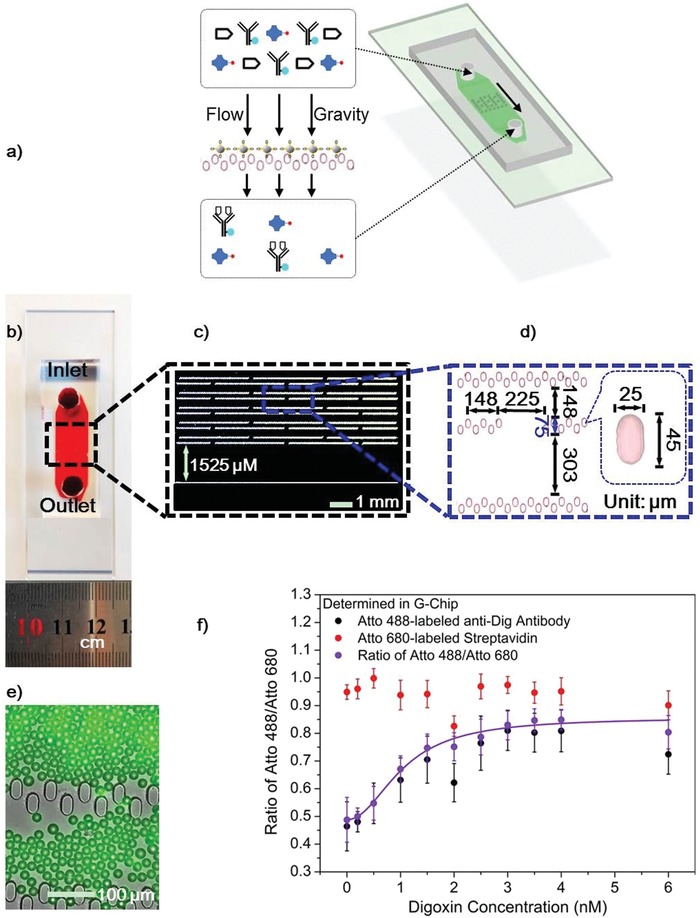
Conceptual view of the G‐Chip and digoxin detection in plasma using the G‐Chip with only 10 µL plasma. a) Schematic view of the designed G‐Chip. b) An optical image of a fabricated chip loaded with red dye. c) Conceptual view of pillar arrays inside the fluid channel. d) Conceptual view and dimensions of the integrated size‐exclusion filters (inset shows the dimensions of the pillars that compose the filters). e) The merged image of bright‐field and fluorescence image of the filters loaded with 20 µm PS–BSA–Digg beads with adsorbed Atto 488‐labeled anti‐Dig antibody. f) Normalized fluorescence intensity of 0.42 nm Atto 488‐labeled anti‐Dig antibody (black dots) and 7.58 nm Atto 680‐labeled streptavidin (red dots) in diluted plasma, and the corresponding signal ratio (purple dots) at various digoxin concentrations. The sample consisting of plasma mixed with labeled antibodies is diluted 1:7 before entering the G‐Chip. However, the concentrations given in (f) are of digoxin in plasma before dilution. All used PS–BSA–Digg beads are recycled beads. Plotted values are mean values with standard deviations (*N* = 3).

To simplify the operational complexity of the assay and to decrease the required blood volume, a miniaturized gravity‐driven microfluidic chip (G‐Chip), which integrates the detection system, has been developed (**Figure** **4**; see Section S8, Supporting Information). Microfluidic chips allow considerable portability and integration, exhibiting great potential for application in point‐of‐care diagnostics and biomedical research.^18^, ^19^, ^20^ However, there are often practical barriers in the form of fluid‐introducing accessories and syringe pump control systems. The present G‐Chip is quite simple and eliminates the use of both centrifuge and syringe pumps to save cost and operational space. The G‐Chip is designed using AutoCAD (Autodesk) and fabricated by photolithography molding techniques (Figure S1 and associated details there, Supporting Information). The G‐Chip is composed of molded polydimethylsiloxane (PDMS), and equipped with two wells, one for plasma inlet and the other for plasma outlet (Figure 4b). The depth of the flow channel in the G‐Chip is ≈40 µm. The width of the channel is 0.9 cm and it contains 11 rows of pillars inside (Figure 4b,c). The whole array of pillars is aligned to the center of the whole channel. In Figure 4c, the dimensions of each pillar and the detailed distribution are shown. It should be noted there are gaps in each row of pillars except in the bottom row, which is continuous. The gap design is made to distribute the beads between neighboring pillars in each row. An actual image of the typical distribution of loaded beads is shown in Figure 4e. Thus, the arrays of integrated pillars made in PDMS efficiently distribute and retain the 20 µm PS–BSA–Digg beads (Figure 4b–e). Since the G‐Chip is driven by gravity, the retention time (and consequently the flow speed through the chip) depends on the tilt angle of the G‐Chip (Section S8, Supporting Information).

When operating the G‐Chip, the chip is first loaded with PS–BSA–Digg beads, which are subsequently retained by the chips‐embedded size‐exclusion filter system. Second, the plasma sample of interest, preincubated with anti‐Dig antibodies, is loaded into the G‐Chip inlet well. All anti‐Dig antibodies that remain with free binding sites will be captured on the PS–BSA–Digg beads. Hereby, anti‐Dig antibodies with free binding sites are retained in the G‐Chip (see Section S8, Supporting Information). For integrated applications of the G‐Chip, the preincubation step (30–40 min) can potentially be circumvented, as no significant difference in the end result was observed when plasma and anti‐Dig antibodies were mixed by simultaneous loading into the inlet well of the G‐Chip (Figure S27, Supporting Information).

Only 10 µL blood plasma is required for analysis in the G‐Chip. First, using a pipette, the 10 µL plasma sample is activated and diluted by mixing with the labeled anti‐Dig antibody and streptavidin probes in 70 µL buffer in the G‐Chip's inlet well (Protocol 2a for the G‐Chip in Methods in the Supporting Information). Second, the chip is tilted with an angle of 30° from the horizontal orientation. Third, after the activated plasma sample has passed through the chip and the nonoccupied antibodies have been captured by the PS–BSA–Digg beads, the solution is collected at the outlet well of the chip. Finally, the samples' fluorescence of the Atto 488‐labeled anti‐Dig antibody and Atto 680‐labeled streptavidin is measured to determine the ratio and in turn the concentration of digoxin (Figure 4f). The end results are quite similar to that monitored in a tube (see Section S8, Supporting Information).

Using a tilt angle of 30° for the G‐Chip, the time for collecting 60 µL activated and filtered plasma in the outlet well is ≈20 min. It could be reduced to 10 min if less liquid is required for the fluorescence measurement. It should be mentioned that the G‐Chip with the captured PS–BSA–Digg beads is continuously recycled by applying the dissociation buffer directly to the chip, thereby liberating the antibodies bound to the PS–BSA–Digg beads captured in the chip (see Protocol to recycle the G‐Chip in Methods in the Supporting Information). Recycled G‐Chips have been applied to generate the concentration regression curve in Figure 4f as well as in all the following experiments. There is no loss of beads or activity when recycling the beads in the chip, since they stay in the filter pillars of the G‐Chip.

### 2.6 Digoxin Concentration Range Detection in Plasma

**Figure 5:**
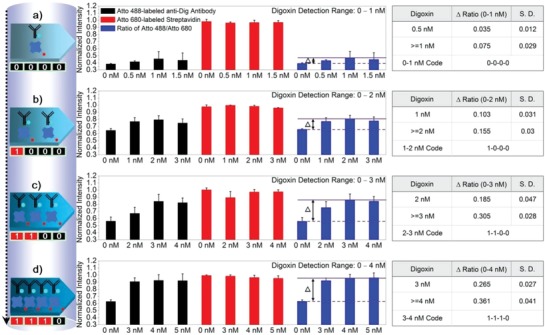
Determination of the digoxin concentration range in plasma with the G‐Chip. System with different probe concentrations (Atto 488‐labeled anti‐Dig antibody/Atto 680‐labeled streptavidin, nm/nm) to detect digoxin at different concentration range in 1:7 diluted plasma with 10 µL plasma: a) 0.14/2.53 to detect digoxin up to 1 nm; b) 0.28/5.05 to detect digoxin up to 2 nm; c) 0.42/7.58 to detect digoxin up to 3 nm; d) 0.56/10.10 to detect digoxin up to 4 nm. Each table summarizes the signal ratio difference to respective background at fixed probe and digoxin concentrations. The codes (a, b, c, d) express the indicated digoxin concentration range in each table. In each symbol in (a, b, c, d), “0” means the obtained signal ratio difference is below the maximum signal ratio difference at the fixed probe concentrations, while “1” represents the signal ratio difference is very close to the maximum signal ratio difference. Plotted values are mean values with standard deviations compared to the mean value (*N* = 3).

In the G‐Chip assay shown in Figure 4, saturation is reached at around 3 nm. The saturation limit is dependent on the concentration of antibody in the assay. For more accurate determination of digoxin concentration in the low nanomolar range, application of more G‐Chips with different antibody concentrations was investigated. When tested at a range of different antibody concentrations, the saturation concentration of digoxin can be determined precisely (Protocol 2b in Methods in the Supporting Information). After optimizing conditions, four successive detection systems are set up to determine concentrations up to 1, 2, 3, and 4 nm digoxin, respectively (**Figure** **5**). If the signal ratio difference between the response and background in each detection system is close to the corresponding maximum value, then the output is defined as “1,” otherwise it is defined as “0.” If two neighboring segments give saturation and unsaturation state, respectively, then digoxin concentration in the sample is located between the neighboring detectable maximum digoxin concentrations. Therefore, a four‐digit code can be set up to determine the concentration range: (0, 0, 0, 0) for 0–1 nm, (1, 0, 0, 0) for 1–2 nm, (1, 1, 0, 0) for 2–3 nm, and (1, 1, 1, 0) for 3–4 nm. It should be noted that only four G‐Chips are prepared to collect data and the prepared G‐Chips are recycled for further use in all the above G‐Chip–associated experiments (Protocol to recycle the G‐Chip in Methods in the Supporting Information). The signal amplitudes and amplitude differences at 0–1 nm are very small and it is difficult to draw clear conclusions from the first series; however for the other concentration ranges, the cutoff values are more distinct and it can, with good certainty, be concluded which of the ranges the concentration belongs to. The most precise concentration determinations in plasma at the low nanomolar range is obtained with the in‐tube detection as shown in Figure 3b, but the four‐chip assay described here also provides readout in the low nanomolar range from the G‐Chip.

## 3 Conclusion

The assay presented here provides a novel method to detect small molecules in plasma using antibodies. It is a type of competitive immunosorption assay, where the presence of the target, here digoxin, binds to the antibody and inhibits further binding. One of the distinct features of the assay is the immobilization of digoxigenin, the aglycon of digoxin, on PS beads linked through BSA. Thus, the excess of the dye‐labeled anti‐Dig is captured on the PS–BSA–Digg beads, whereas the dye‐labeled antibodies that are occupied by digoxin remain in solution after centrifugation or filtration. The fluorescence from the solution is therefore proportional to the concentration of digoxin. One drawback of the method is that it is highly reliable on the quality of the antibody and a certain background from nonbinding antibody is observed in the experiments. The background was constant of ≈40% during experiments. This is probably induced by loss of activity of the antibody in global labeling, and possibly, it may be improved by site‐specific labeling.^21^, ^22^

Another asset of the method is that dye‐labeled streptavidin is used as a reference to level the variation. By using the ratio between the fluorescence from Atto 488‐labeled anti‐Dig antibody and Atto 680‐labeled streptavidin as the readout signal, it is observed that error bars are largely reduced. Just like ratiometric sensing, the present strategy takes advantage of this calibration for reducing the influence of system fluctuations in parallel operations, environmental conditions, and excitation intensity by adding a reference. Therefore, high robustness, reliability, and reproducibility in this two signal‐mode strategy are observed. This assay is applied to detection of digoxin in plasma and provides highly sensitive and selective readout with a detection limit of 0.2 nm and detection range of 0.2–6 nm, which are within the range for clinical monitoring of digoxin.

The assay is integrated on the G‐Chip which is driven by gravity and no pump system is required. The low amount of plasma required for the G‐Chip enables detection from a drop of blood from a fingerstick. Accordingly, a new protocol requiring only 10 µL plasma is designed. The integrated filter system retains the PS–BSA–Digg beads in the chip and thereby centrifugation of the beads is avoided. Finally, based on the G‐Chip, a strategy is provided for detection of digoxin in low concentration range, where the sample is located. In addition, five different protocols are set up for real sample detection with the use of plasma.

Furthermore, the PS–BSA–Digg beads are recyclable and their activity is retained by flushing the beads for short time with a dissociation buffer. Most importantly, the beads can be recycled once retained in the G‐Chip simply by incubating the chip with the dissociation buffer. The integration of the assay in a microfluidic chip and the recyclability of the device hold great potential for reducing the costs in potential practical applications.

We have developed the assay for quantification of digoxin in plasma, and it is a difficult target since it has a low therapeutic range of 0.6–2.6 nm. However, the method is generic and it should be straightforward to extend it to other small‐molecule drugs as long as they can be immobilized on the PS beads and an antibody for the drug has been developed. Multiplex detection of more small molecules of interest may also be developed,^8^, ^23^, ^24^, ^25^, ^26^, ^27^, ^28^ if different antibodies with distinguishable dyes are applied in the same tube or G‐Chip. The precision and reliability of the method and the integration of the method into the G‐Chip hold potential for development of a point‐of‐care technology based on the presented method.^29^

## 4 Experimental Section

*Materials*: All chemicals used were of analytical grade and were used without further purification. Functionalized PS beads (Polybead Carboxylate Microspheres 20 µm, 2.5 solids w/v) and PolyLink Protein Coupling Kit were purchased from Polysciences, Inc. (Warrington, PA). Silicon wafers (4 in. were purchased from Corning Inc. (Corning, NY). SU‐8 3050 photoresist and SU‐8 developer were purchased from MicroChem Corp. (Newton, MA). PDMS (RTV615) was purchased from Momentive Performance Materials (Waterford, NY). All devices were designed as computer graphics using AutoCAD software and then printed out as 10 µm resolution film masks by JD Photo Data (Herts, UK). BSA, Atto 680‐labeled streptavidin, polyclonal anti‐Dig antibody from sheep, Atto 488 labeling kit, digoxigenin NHS‐ester, PBS tablet, antibody stabilizer, small molecules, and other associated materials were all purchased from Sigma‐Aldrich. The water used throughout all experiments was purified through a Milli‐Q Biocell System. Organic reactions were monitored by thin‐layer chromatography. Polyacrylamide gels were stained with SimplyBlue SafeStain (Life Technologies) according to manufactures protocol. Antigen–antibody dissociation kit was purchased from LaboratoryEssentials (BioWORLD, Dublin, USA). Refer to the Supporting Information for detailed list of instruments.

*Methods*: Preparation of PS–BSA–Digg Beads. Conjugation of Digoxigenin NHS‐Ester to BSA: Digoxigenin NHS‐ester (1 mg, 1.52 µmol) was dissolved in DMSO (1 mL), divided into 10 aliquots, freeze‐dried, and stored at −20 °C. One aliquot was dissolved in 100 µL DMSO immediately before use. Digoxigenin NHS‐ester (90 µL, 1.52 mm) and BSA (60 µL, 1%) were added into K_2_HPO_4_ (135 µL, 0.2 m, pH: 9.1). The mixture was kept at 4 °C in a refrigerator overnight. Then, washing buffer (1.53 mL, 0.025 m KH_2_PO_4_, 0.15 m NaCl, 0.01% NaN_3_, pH: 7.2) was added in and separated with an Amicon filter (15 000 × g, 5 min, for separation twice and then 2000 × g, 4 min, for collection). The final volume of collected conjugation sample was about 90 µL. The conjugation was confirmed with polyacrylamide gel electrophoresis (PAGE) analysis and the yield was about 85% in comparison with control experiments with only the same amount of BSA.

*Coupling of BSA‐Digg Conjugates with Carboxylate‐Functionalized PS Beads*: The above‐obtained BSA‐Digg conjugates (100 µL) were diluted with BSA (about 100 µL, 1%), and PS beads (about 900 µL) were coupled according to the procedure in the PolyLink Protein Coupling Kit. After centrifugation, the modified beads were suspended in 1.5 mL washing/storage buffer. The final concentration was about 1.5% solid w/v.

*Fabrication of the G‐Chip*: The microfluidic structure was designed with AutoCAD (Autodesk). The G‐Chip was fabricated by the use of soft lithography and PDMS molding technique.

Refer to the details of associated methods in the Supporting Information, including details to fabricate microfluidic chips, native polyacrylamide gel electrophoresis, and associated protocols.

## Conflict of Interest

The authors declare no conflict of interest.

## Supporting information

Supplementary
