# Supplementary material for: Quantitative Detection of Digoxin in Plasma Using Small‐Molecule Immunoassay in a Recyclable Gravity‐Driven Microfluidic Chip
Source: Adv Sci (Weinh). 2019 Jan 27;6(6):1802051. doi: 10.1002/advs.201802051 (PMC6425438; doi:10.1002/advs.201802051)
Supplement: Supplementary file 1 — Supplementary [file ADVS-6-1802051-s001.pdf]

Copyright WILEY-VCH Verlag GmbH & Co. KGaA, 69469 Weinheim, Germany, 2019.

ADVANCED SCIENCE
Open Access

Supporting Information

for *Adv. Sci.,* DOI: 10.1002/advs.201802051

Quantitative Detection of Digoxin in Plasma Using Small-Molecule Immunoassay in a Recyclable Gravity-Driven Microfluidic Chip

Hailong Li, Jesper Vinther Sørensen, and Kurt Vesterager Gothelf**

Copyright WILEY-VCH Verlag GmbH & Co. KGaA, 69469 Weinheim, Germany, 2018.

Supporting Information

Quantitative Detection of Digoxin in Plasma using Small-molecule Immunoassay in a Recyclable Gravity-driven Microfluidic Chip

Hailong Li,* Jesper Vinther Sørensen, and Kurt Vesterager Gothelf*

Materials.
All chemicals used were of analytical grade and were used without further purification. Functionalized polystyrene beads (Polybead® Carboxylate Microspheres 20 µm, 2.5 solids w/v) and PolyLink Protein Coupling Kit were purchased from Polysciences, Inc. (Warrington, PA). Silicon wafers (4-inch were purchased from Corning Inc. (Corning, NY). SU-8 3050 photoresist and SU-8 developer were purchased from MicroChem Corp. (Newton, MA). Polydimethylsiloxane (PDMS RTV615) was purchased from Momentive Performance Materials (Waterford, NY). All devices were designed as computer graphics using AutoCAD software and then printed out as 10-µm-resolution film masks by JD Photo Data (Herts, UK). Bovine serum albumin, Atto 680-labeled streptavidin, polyclonal anti-Dig antibody from sheep, Atto 488 labeling kit, digoxigenin NHS-ester, PBS tablet, antibody stabilizer, small molecules, and other associated materials were all purchased from Sigma-Aldrich. The water used throughout all experiments was purified through a Milli-Q Biocell System. Organic reactions were monitored by thin-layer chromatography (TLC). Polyacrylamide gels were stained with SimplyBlue^{TM} SafeStain (Life Technologies^{TM}) according to manufactures protocol. Antigen-antibody dissociation kit was purchased from LaboratoryEssentials (BioWORLD, Dublin, USA).

Instruments.
Fluorescence intensities were recorded on a Fluoromax-3 spectrofluorometer (Horiba Jobin Yvon, Longjumeau, France) using a Hellma Quartz Suprasil 3 mm x 3 mm, 60 µL cuvette. After electrophoresis, the gel was scanned by a Typhoon scanner (Amersham Biosciences). Gel electrophoresis was performed with a PowerPac^{TM} Basic Power Supply and Criterion^{TM} Vertical Electrophoresis Cell (Bio-Rad, Denmrk). Aligament and UV exposure of resist coated wafers were done on EVG®610 mask aligner (EVG, St. Florian am Inn, Austria). Plasma treatment is performed on reactive ion etcher (RIE) (Plasmalab 80 Plus, Oxford Instruments, Oxfordshire, UK). Optical images of fabricated molds on wafer were taken on Zeiss Axioscope A1 optical microscope (Carl Zeiss Microscopy Ltd, Cambridge, UK). The height of the microstructure on wafer was measured with Brucker DektakXT stylus profiler (Tucson, AZ, USA).

Native Polyacrylamide Gel Electrophoresis.
Polyacrylamide gels (6%) were prepared with Tricine (1 mL, 500 mM), acrylamide (1.5 mL, 40%), and water (7.5 mL). Then ammonium persulfate (APS) (100 µL, 10%) and tetramethylethylenediamine (TEMED) (10 µL) were added and allowed to polymerize at least 30 minutes before use. The gel was run at a constant voltage of 100 V in 50 mM Tricine as running buffer over a period of about 2.5 h.

Fabrication of the G-Chip.
The microfluidic structure was designed with AutoCAD (Autodesk). The G-Chip was fabricated by the use of soft lithography and polydimethylsiloxane (PDMS) molding technique. The dimensions are shown in **Figure** S1.

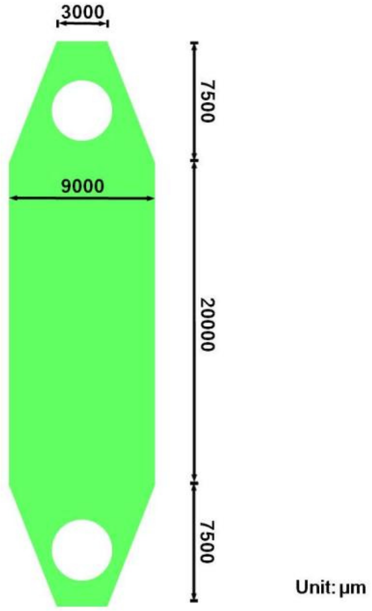

Figure S1 The dimensions of G-Chip outline sketch.

To achieve the desired height of the mold layer at about 30-40 μm, negative photoresist SU8-3050 was spun on the silicon wafer at 500 rpm for 10 seconds with an acceleration of 100 rpm/s to spread out the photoresist, then at 3000 rpm for 30 seconds with an acceleration of 100 rpm/s. Then 30 seconds were set up to stop. The coated wafer was then baked at 95 °C for 15 minutes. After cooling down, the wafer was patterned with UV light in the photomask aligner with an exposure dose of 150-250 mJ/cm^2, followed by immediate postexposure baking at 65 °C for 1 minutes first and then at 95 °C for 5 minutes. Then, it was developed in SU-8 developer for 10 minutes and washed with isopropyl alcohol. After developing, the wafer was hard-baked at 140 °C for 1 hour. After being treated with an anti-adhesive agent, trimethylchlorosilane (TMCS), via vapor at reduced pressure for 30 minutes, the wafer mold was casted with pre-polymer of RTV 615 PDMS part A and B (10:1), and then baked at 75 °C overnight. The PDMS layer was peeled off the mold, cut, and punched for inlet and outlet holes. It was further chemically bonded to a glass slide after oxygen plasma treatment. The fresh G-Chip was treated with detection buffer overnight. Then PS-BSA-Dig beads of the desired amount were loaded onto the embedded filters driven by gravity.

Methods

Labeling anti-Dig Antibody with Atto 488
The labeling is done following the protocol in the labeling kit from Sigma-Aldrich. Briefly, anti-Dig antibody (200 µL, 0.5 mg/mL) was mixed with reaction buffer (25 µL) by pipetting up and down several times. Then the solution is transferred to the vial containing dye (the amount is suitable to label 50-100 µg antibody) and incubated in the dark for 30 minutes. The solution is now ready to use. The labeled antibody is diluted with antibody stabilizer (1 mL).

Preparation of PS-BSA-Digg Beads
Digoxigenin NHS-ester (1 mg, 1.52 µmol) was dissolved in DMSO (1 mL), divided into 10 aliquots, freezed-dried, and stored at -20 °C. One aliquot was dissolved in 100 µL DMSO immediately before use.
Conjugation of digoxigenin NHS-ester to BSA. Digoxigenin NHS-ester (90 µL, 1.52 mM) and BSA (60 µL, 1%) were added into K_2HPO_4 (135 µL, 0.2 M, pH: 9.1). The mixture was kept at 4 °C in a refrigerator overnight. Then, washing buffer (1.53 mL, 0.025 M KH_2PO_4, 0.15 M NaCl, 0.01% NaN_3, pH: 7.2) was added in and separated with an Amicon filter (15000 g 5 min for separation twice and then 2000 g 4 min for collection). The final volume of collected conjugation sample is about 90 µL. The conjugation is confirmed with PAGE analysis (**Figure S2**) and the yield is about 85% in comparison with control experiments with only the same amount of BSA.

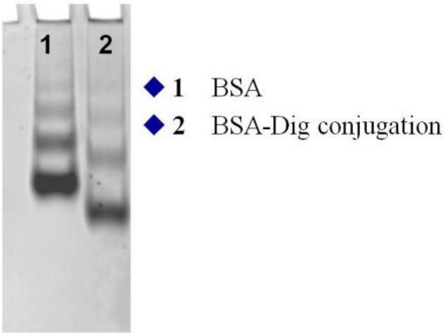

Figure S2 Native 6% PAGE analysis of BSA and BSA-Digg conjugation (both are around 0.03 µg). Lane 1: BSA. Lane 2: Products from BSA and Digoxigenin NHS-ester reaction.

Coupling of BSA-Dig Conjugates with Carboxylate-functionalized PS Beads. The above-obtained BSA-Dig conjugates (100 µL) were diluted with about BSA (100 µL, 1%), and PS beads (about 900 µL) were coupled according to the procedure in the PolyLink Protein Coupling Kit. After centrifugation, the modified beads were suspended in 1.5 mL washing/storage buffer. The final concentration is about 1.5% solid w/v. **Figure S3** shows the bright-field and fluorescence image of PS-BSA-Digg beads after incubation with Atto 488-labeled anti-Dig antibody.

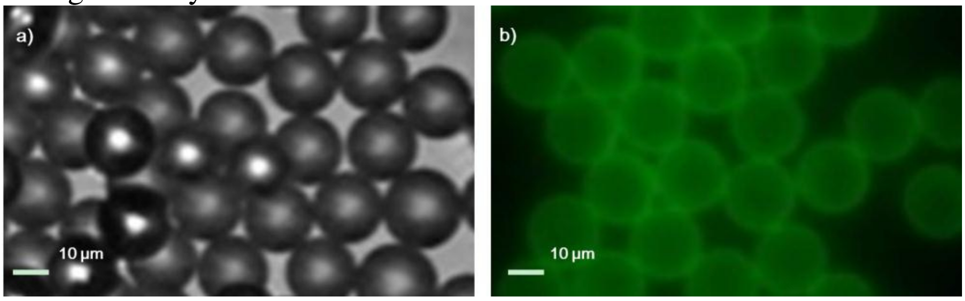

Figure S3 a) Bright-field and b) fluorescence image of PS-BSA-Digg beads after incubation with Atto 488-labeled anti-Dig antibody.

Spectrofluorimetry Studies
The desired concentrations of Atto 488-labeled anti-Dig antibody and Atto 680-labeled streptavidin were obtained by diluting the corresponding stock solution in antibody stabilizer with detection buffer (PBS buffer with 0.1% BSA, 0.05% Tween-20, and 0.02% NaN_3). The total volume of each sample is 200 μL. The volume of each sample for fluorescence measurement is 70 μL. To monitor Atto 488 fluorescence, excitation was carried out at 480 nM, and emission intensity was monitored at around 520 nM. For Atto 680 fluorescence, excitation was carried out at 670 nM, and emission intensity was monitored at around 700 nM. In the above fluorescence measurement, slit widths for both excitation and emission were set at 5 nM. In normalizing the fluorescence intensity, the fluorescence intensities from control blank sample at fixed probe concentrations were set to 1. Error bars represent the standard deviation of at least three independent experiments.

Protocols

Protocol 1a for Digoxin Detection in a Tube**.**
1. Plasma is diluted with detection buffer (1:2).
2. In a total volume of 200 μL, the stock solution of protein probes is added in to achieve desired probe concentrations in diluted plasma (Atto 488-labeled anti-Dig antibody: 1.11 nM; Atto 680-labeled streptavidin: 20.20 nM). The mixture is gently shaken on a rotator for about 30-40 minutes.
3. About 3.5 μL PS-BSA-Digg beads (recycled beads here) are added in and the mixture is kept shaking for another 30-40 minutes. Then the beads are centrifuged down at 3000 rpm over 5-10 minutes. From the supernatant, 70 μL is drawn to measure the fluorescence intensity of Atto 488-labeled anti-Dig antibody and Atto 680-labeled streptavidin. Then the intensity is normalized according to a control blank sample with only probes and the signal ratio of Atto 488/Atto 680 is calculated.
4. The digoxin concentration C in pure plasma is determined according to the concentration regression curve (**Figure 3a**).

Protocol 1b for Digoxin Detection in a Tube**.**
1. Plasma is diluted with detection buffer (1:3).
2. In a total volume of 200 μL, the stock solution of protein probes is added to the plasma to achieve the desired probe concentrations in diluted plasma (Atto 488-labeled anti-Dig antibody: 3.33 nM; Atto 680-labeled streptavidin: 60.61 nM). The mixture is gently shaken on a rotator for about 30-40 minutes.
3. PS-BSA-Diggg beads (10 μL, recycled beads here) are added and the mixture is kept shaking for another 30-40 minutes. Then the beads are centrifuged down at 3000 rpm over 5-10 minutes. From the supernatant, 70 μL is drawn to measure the fluorescence intensity of Atto 488-labeled anti-Dig antibody and Atto 680-labeled streptavidin. Then the intensity is normalized according to a control blank sample with only probes and the signal ratio of Atto 488/Atto 680 is calculated.
4. The digoxin concentration C in the diluted plasma is determined according to the concentration regression curve (**Figure S21b**). The digoxin concentration in pure plasma is 4×C.

Protocol 1c for Digoxin Detection in a Tube**.**
1. Plasma (10 μL) is diluted with detection buffer (1:7).
2. An aliquot from the stock solution of protein probes is added in to achieve desired probe concentrations in diluted plasma (Atto 488-labeled anti-Dig antibody: 0.42 nM; Atto 680-

labeled streptavidin: 7.58 nM). The mixture is gently shaken on a rotator for about 30-40 minutes.
3. Diluted PS-BSA-Diggg beads (2 µL, 1:4 diluted, recycled beads here) are added in and the mixture is kept shaking for another 30-40 minutes. Then the beads are centrifuged down at 3000 rpm over 5-10 minutes. A volume of 65 µL supernatant is drawn to measure the fluorescence intensity of Atto 488-labeled anti-Dig antibody and Atto 680-labeled streptavidin. Then the intensity is normalized according to a control blank sample with only probes and the signal ratio of Atto 488/Atto 680 is calculated.
4. The digoxin concentration C in pure plasma is determined according to the concentration regression curve (**Figure S28b**).

Protocol 2a for Digoxin Detection in the G-Chip**.**
1. Plasma (10 µL) is diluted with detection buffer (1:7).
2. The stock solution of protein probes is added in to achieve desired probe concentrations in diluted plasma (Atto 488-labeled anti-Dig antibody: 0.42 nM; Atto 680-labeled streptavidin: 7.58 nM). The mixture is directly loaded into the inlet well of the G-Chip at fixed tilt angle of 30°.
3. From the outlet well, 70 µL is collected over a period of about 25 minutes to fix the setup for fluorescence measurement (less time is needed to collect liquids if the chosen fluorescence measurement requires a lower volume). Then the intensity is normalized according to a control blank sample with only probes and the signal ratio of Atto 488/Atto 680 is calculated.
4. The digoxin concentration C in pure plasma is determined according to the concentration regression curve (**Figure 4f**).

Protocol 2b for digoxin Detection Range in G-Chip**.**
1. Plasma (10 µL) is diluted with detection buffer (1:7). Four experiments are performed in parallel.
2. The stock solution of protein probes is added in to achieve desired probe concentrations in diluted plasma (Atto 488-labeled anti-Dig antibody/Atto 680-labeled streptavidin: nM/nM): (**a**) 0.14/2.53 for the first G-Chip; (**b**) 0.28/5.05 for the second G-Chip; (**c**) 0.42/7.58 for the third G-Chip; (**d**) 0.56/10.11 for the fourth G-Chip. Then, the mixtures are directly loaded into the inlet well of corresponding G-Chips at fixed tilt angle of 30°, respectively.
3. The liquid is collected in the outlet well. Here we choose to collect about 70 µL over a period of about 25 minutes to fix the setup for fluorescence measurement (It needs less time to collect liquids if your chosen fluorescence measurement needs less sample). Then the intensity is normalized according to a control blank sample with only probes and the signal ratio of Atto 488/Atto 680 is calculated.
4. The signal ratio difference from each G-Chip is calculated and the relative ration amplitudes is compared.
5. The concentration range where digoxin concentration in pure plasma is located according to the code in **Figure S30** is determined.

Protocol to recycle the G-Chip**.**
1. The recycled G-Chip is ished twice with 50 µL water twice.
2. The dissociation buffer (50 µL) is loaded into the G-Chip. The G-Chip is kept at a 30° tilt angle for 10 minutes, and it is then positioned horizontal for 15-20 minutes.
3. The G-Chip is then ished with 50 µL water twice, and 50 µL detection buffer once.
4. The final recycled G-Chip is kept with its channel flooded with detection buffer until next use.

Prior to next use: All the liquid in both the inlet and outlet is removed before loading for detection.

Results and Discussion

Section S1 Investigation of the stability of the two signal-mode strategy

To examine the robustness and reliability of this two signal-mode assay, the fluorescence signals at different probe concentrations were measured at different times over the course of three days and the signal ratios were calculated. As shown in **Figure S4a**, the error bars from the individual signals, from either Atto 488-labeled anti-Dig antibody or Atto 680-labeled streptavidin, are much larger than that in the corresponding signal ratios, as verified at four different probe concentrations. All signals are also categorized in groups of different days at different probe concentrations. **Figure S4b** shows the summarized distribution and variation from day-to-day comparison. In general, the distribution range in the normalized intensity (dI, given by Eq S1) from single signals at the same day is 0.060-0.262 for Atto 488-labeled anti-Dig antibody and 0.063-0.250 for Atto 680-labeled streptavidin, respectively.

$$dI = \text{Normalized maximum intensity } (I_{max}) - \text{Normalized minimum intensity } (I_{min}) \qquad (S1)$$

However, the distribution range for the signal ratio of Atto 488/Atto 680 with normalized intensity is between 0.014-0.097, which is much narrower relative to the individual dI values of Atto 488 and Atto 680 although the ratio slightly increases at low probe concentration. In addition, all the P values are calculated for day-to-day comparisons. All the P values from signal ratio comparison are equal to or above 0.05, which demonstrates that the results from two signal-mode strategy are reproducible and independent of time of measurement. The signals in **Figure S4a** are also categorized in groups for sample-to-sample and concentration-to-concentration comparisons, respectively (Supporting **Figure S5**, **S6**, **Table S1**, and associated discussion there), further confirming the advantage.

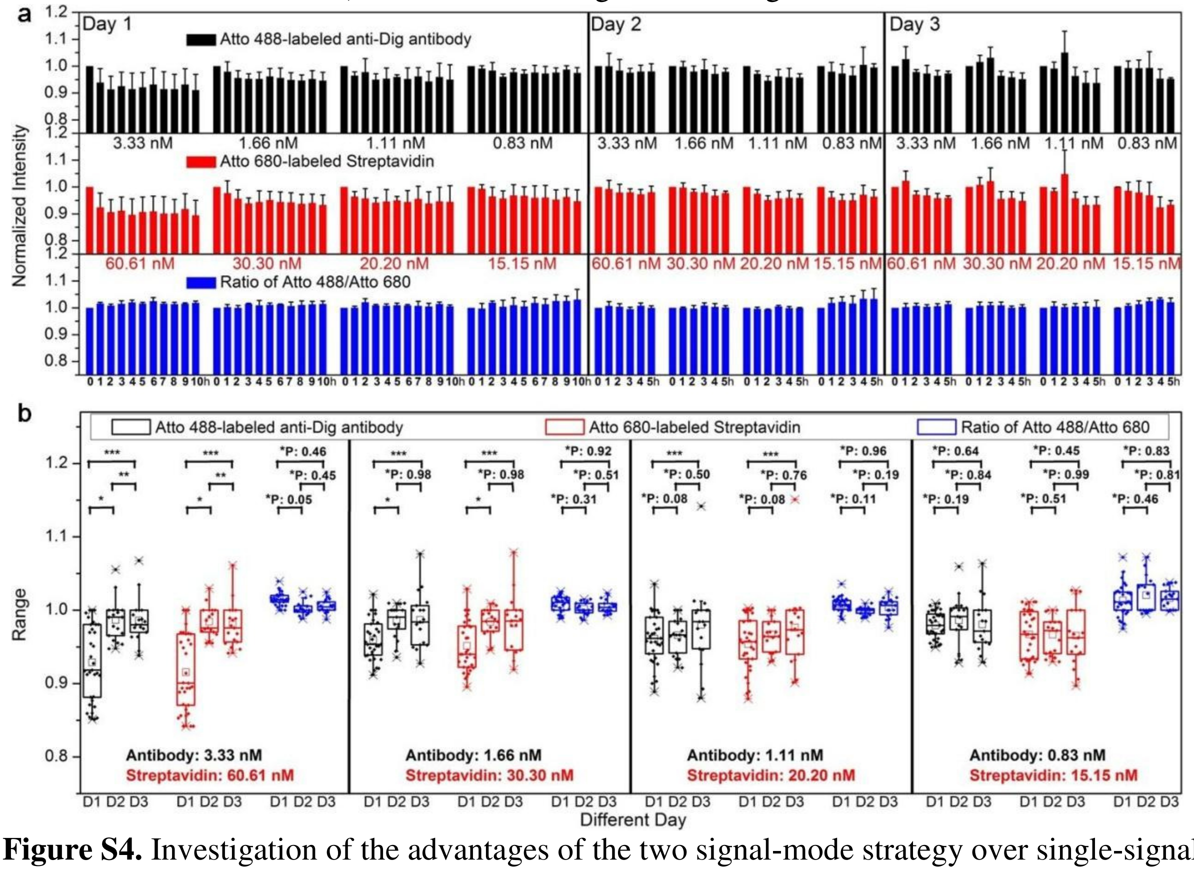

Figure S4. Investigation of the advantages of the two signal-mode strategy over single-signal mode measurements. (**a**) Normalized fluorescence intensity of Atto 488-labeled anti-Dig antibody and Atto 680-labeled streptavidin at different probe concentrations, and the corresponding Atto 488/Atto 680 signal ratio. Fluorescence signals are measured over three

days: 0-10 h on Day 1, 0-5 h on Day 2, and 0-5 h on Day 3. (**b**) Box and whisker plots of the normalized fluorescence intensity and corresponding signal ratio in (a). Horizontal lines are medians, boxes show the interquartile range (IQR), error bars show the full range excluding outliers (crosses) defined as being more than ±1.5IQR outside the box. Day-to-day variation is also compared at different probe concentrations. Asterisks indicate statistically significant differences ($P < 0.05$) in Day-to-Day variance; P values are also given where there are no significant differences ($P > 0.05$). Probe concentrations (Atto 488-labeled anti-Dig antibody/Atto 680-labeled streptavidin, nM/nM): 3.33/60.61, 1.66/30.30, 1.11/20.20, 0.83/15.15. Plotted values are mean values with standard deviations compared to the mean value (N=3).

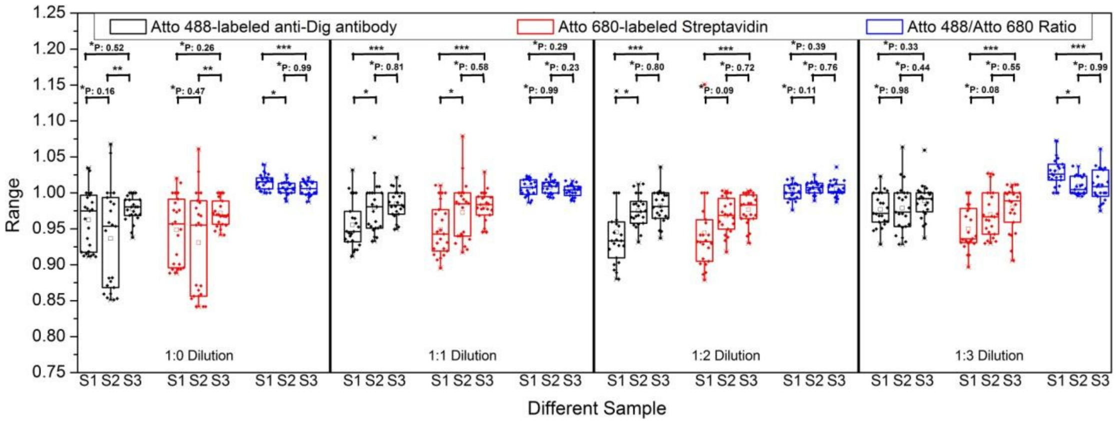

Figure S5. Box and whisker plots of the normalized fluorescence intensity and signal ratio from **Figure S4a** at different probe concentrations categorized in terms of the different samples. Horizontal lines are medians, boxes show the interquartile range (IQR), error bars show the full range excluding outliers (crosses) defined as being more than ±1.5IQR outside the box. Day-to-day variation is also compared at different probe concentrations. Asterisks indicate statistically significant differences ($P < 0.05$) in Sample-to-Sample variance, P values are also given where there is no significant differences ($P > 0.05$). 1:0 Dilution, 1:1 Dilution, 1:2 Dilution, and 1:3 Dilution refer to probe concentrations (Atto 488-labeled anti-Dig antibody/Atto 680-labeled streptavidin, nM/nM): 3.33/60.61, 1.66/30.30, 1.11/20.20, 0.83/15.15, respectively.

Discussion of **Figure S5**

After categorizing the signal and signal ratios of the same sample at different concentrations for sample-to-sample comparison, the variance between single-mode signals, either from Atto 488-labeled antibody or Atto 680-labeled streptavidin, is much larger than signals ratios of Atto 488/Atto 680. Accordingly, the plot distribution of signal ratios is much narrower than single signals. In general, the distribution range (Maximum - Minimum) from single signals of different samples is about 0.065-0.225 for Atto 488-labeled anti-Dig antibody and about 0.062-0.225 for Atto 680-labeled streptavidin, respectively. However, the distribution range for the signal ratio of Atto 488/Atto 680 is between about 0.027-0.093, which is much narrower than that from single signals although it becomes a little larger at low probe concentration (1:3 Dilution). In addition, all the P values were calculated for Sample-to-Sample comparison. The number of $P > 0.05$, which means there is no significant difference, is 8 for signal ratios (about 67% in total times of comparison), while the number from either single signal is 7 (about 58%). This result confirms the high reproducibility of the suggested two signal-mode strategy no matter what concentrations the probes are set.

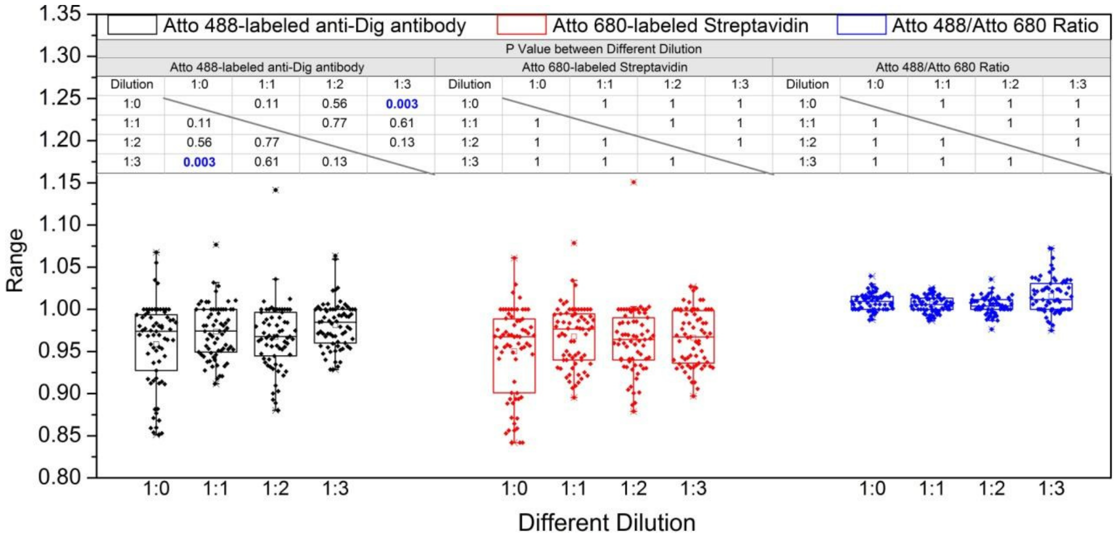

Figure S6. Box and whisker plots of the normalized fluorescence intensity and signal ratio from **Figure S4a** categorized in terms of different probe concentrations (achieved by different dilutions). Horizontal lines are medians, boxes show the interquartile range (IQR), error bars show the full range excluding outliers (crosses) defined as being more than ±1.5IQR outside the box. Day-to-day variation is also compared at different probe concentrations. Asterisks indicate statistically significant differences ($P < 0.05$) in Concentration-to-Concentration variance, P values are also given where there is no significant differences ($P > 0.05$). 1:0 Dilution, 1:1 Dilution, 1:2 Dilution, and 1:3 Dilution refer to probe concentrations (Atto 488-labeled anti-Dig antibody/Atto 680-labeled streptavidin, nM/nM): 3.33/60.61, 1.66/30.30, 1.11/20.20, 0.83/15.15, respectively.

Discussion of **Figure S6**

All P values were also calculated by categorizing results from samples in terms of different concentrations. Only one P value is below 0.05 (indicated with bold blue font in the inserted table), which means there is significant difference. Similarly, the variance from single signals is much larger than the signal ratios and the plot distribution of signal ratios is much narrower. In spite of this, the concentration of probes on the signals and signal ratios do not influence the two signal-mode strategy much. The plot distributions from either single signals or signal ratios are similar, respectively, although the signal ratio variance at low probe concentrations (1:3 diluted) becomes a little larger. The high robustness of the two signal-mode strategy is further confirmed, demonstrating its great advantage and potential to develop or improve a detection method.

Table S1 Concentration-to-concentration comparison.

P Value between Different Dilution												
	1:0 Dilution			1:1 Dilution			1:2 Dilution			1:3 Dilution		
	Atto 488	Atto 680	Ratio	Atto 488	Atto 680	Ratio	Atto 488	Atto 680	Ratio	Atto 488	Atto 680	Ratio
Sample 1: Day 1-2	*	*	0.09	0.07	*	*	0.98	0.57	0.06	*	0.1	0.92
Sample 1: Day 1-3	***	***	0.79	***	***	0.29	0.91	0.15	***	0.95	0.74	0.33
Sample 1: Day 2-3	0.93	0.71	0.26	0.64	0.98	**	0.85	0.69	0.14	**	0.43	0.25
Sample 2: Day 1-2	*	*	0.17	*	*	0.61	0.06	*	0.08	0.25	0.83	*
Sample 2: Day 1-3	***	***	0.52	***	***	***	***	***	0.39	***	***	0.9
Sample 2: Day 2-3	0.59	0.72	0.72	0.9	0.48	**	0.36	1	**	**	**	0.13
Sample 3: Day 1-2	*	*	0.14	0.49	0.6	0.43	0.11	0.14	0.46	0.8	0.09	*
Sample 3: Day 1-3	***	***	0.05	0.92	0.6	0.21	0.49	0.21	0.93	***	***	***
Sample 3: Day 2-3	**	**	0.84	0.3	0.16	0.87	0.67	0.98	0.29	**	0.1	0.79
Mean: Day 1-2	*	*	*	0.37	*	0.29	0.94	0.59	*	0.66	1	0.47
Mean: Day 1-3	***	***	0.18	0.33	***	0.92	0.35	0.24	0.89	0.98	0.98	0.87
Mean: Day 2-3	0.99	0.97	0.54	1	0.98	0.48	0.62	0.82	0.05	0.82	0.99	0.82

Table S1. P values calculated by comparing signals from the same sample measured from different days. Mean value of signals from samples at the same concentration was also

calculated to compare Day-to-Day variance. Asterisks indicate statistically significant differences ($P < 0.05$) in Day-to-Day variance from the same sample, P values are also given where there is no significant differences ($P > 0.05$). 1:0 Dilution, 1:1 Dilution, 1:2 Dilution, and 1:3 Dilution refer to probe concentrations (Atto 488-labeled anti-Dig antibody/Atto 680-labeled streptavidin, nM/nM): 3.33/60.61, 1.66/30.30, 1.11/20.20, 0.83/15.15, respectively.

Discussion of **Table S1**

All P values were also calculated by comparing the same sample at different concentration to compare day-to-day variance. The mean values of samples at the same concentration were also compared to show day-to-day variance. From the results, about 77% of the P values from signal ratio comparison is above 0.05 (37 times out of total 48 times of comparison), which means there is no significant difference between signal ratios of the same sample in day-to-day variance. However, from comparisons of single-mode signals, the percent of $P > 0.05$ is about 60% for Atto 488-labeled anti-Dig antibody (29 times out of total 48 times of comparison) and about 58% for Atto 680-labeled streptavidin (28 times out of total 48 times of comparison). And almost all the P values from comparisons between mean values of signal ratios are above 0.05. This result also confirms the great advantages of two signal-mode strategy in high reproducibility, stability, robustness, and reliability to develop a detection method of high precision.

Section S2 Detection Feasibility Test

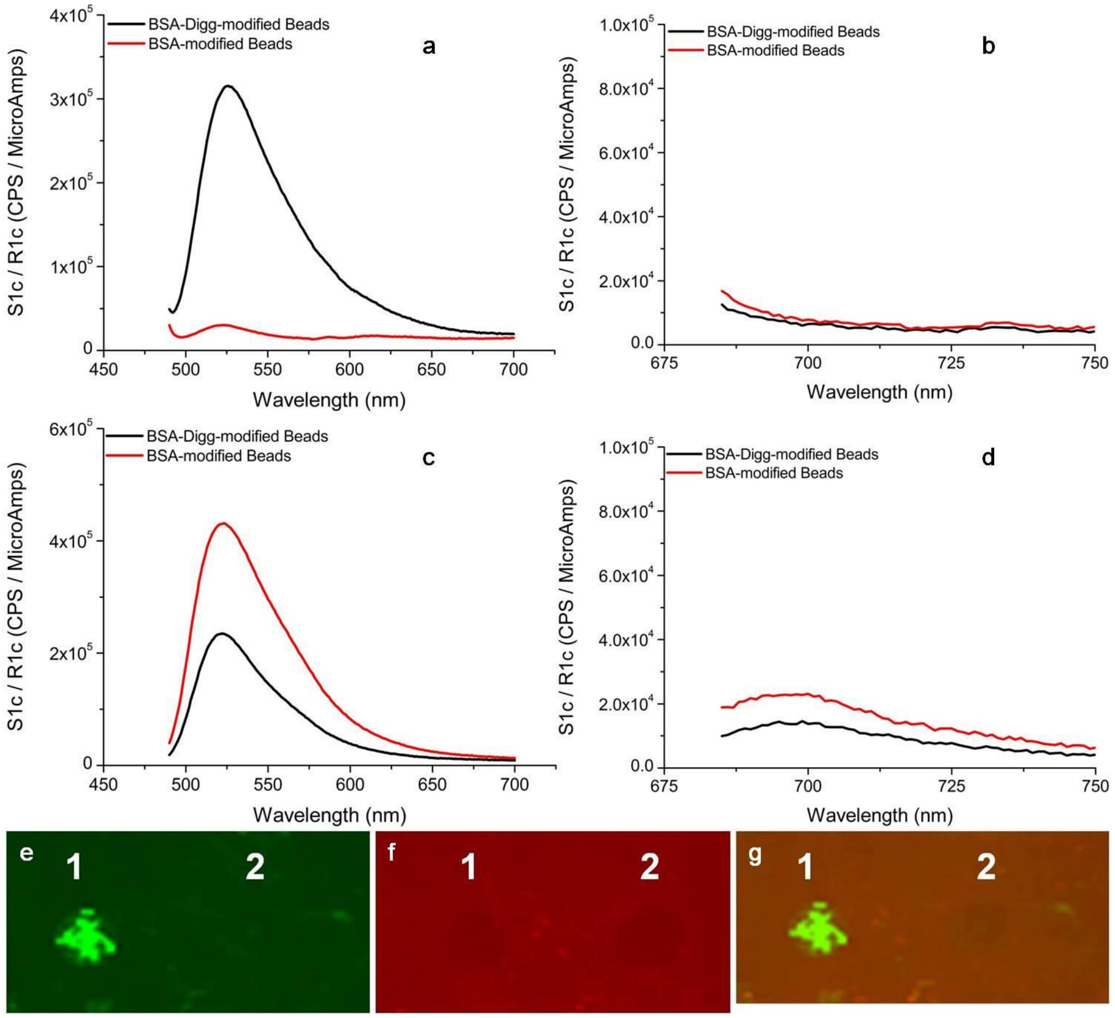

Figure S7. Feasibility of digoxin detection with the use of two signal-mode strategy. (a) and (b) are fluorescence emission spectra of Atto 488-labeled anti-Dig antibody and Atto 680-labeled streptavidin, respectively, after centrifugation and resuspension of PS-BSA-Digg beads and PS-BSA control beads in fresh buffer. (c) and (d) are fluorescence emission spectra of Atto 488-labeled anti-Dig antibody and Atto 680-labeled streptavidin from the supernatant after centrifugation of PS-BSA-Digg beads and PS-BSA control beads, respectively. (e) and (f) are fluorescence images of Atto 488-labeled anti-Dig antibody and Atto 680-labeled streptavidin by dropping 2 μL beads residue on a thin transparent film scanned on Typhoon scanner (Amersham Biosciences): 1, PS-BSA-Digg beads; 2, PS-BSA beads. (g) shows the emerged picture of (e) and (f).

Section S3 Investigation of detection condition, kinetics, and digoxin detection in pure buffer

After confirming the feasibility for digoxin detection, a range of probe concentrations is set up to cover the relevant digoxin concentration range for clinical requirements (data not shown). With the optimized probe concentrations, the optimal amount of PS-BSA-Digg beads for the system is found. In the absence of digoxin, a series of experiments with variable amount of beads are prepared to identify the optimal amount of modified beads which result in the lowest background signal in detection buffer. As shown in **Figure S8**, the fluorescence intensity from Atto 488-labeled anti-Dig antibody in the supernatant decreases with the increment of the amount of PS-BSA-Digg beads while the fluorescence intensity from Atto 680-labeled streptavidin approximately remains constant. The signal ratio of Atto 488/Atto 680 follows the same trend as Atto 488. As a function of the amounts of modified beads, the signals on the modified beads, calculated through a simple conversion of $(1 - {}^{I_{Atto\,488}}/_{I_{Atto\,680}})$, can be simulated well with the Langmuir-Freundlich equation, which is the exponential Langmuir model (see the inset in **Figure S8c**, and **Table S2** for detailed information of all simulations in this paper).[1] The signals are almost identical and non-zero as the amount of beads is increased from 10 μL to 12 μL. The remaining background signals may arise from inactive dye-labeled antibody. Based on the results, 10 μL PS-BSA-Digg beads are chosen as the optimal amount for further experiments under this probe concentration. The kinetics for the immunoadsorption is further investigated (Supporting **Figure S9** and **S10**). It takes about 60 min to achieve almost full balance. However, 20-30 min is enough to reach 95% sorption of that in full balance. After calculating the signals from the supernatant to the modified beads with the simple conversion of $(1 - {}^{I_{Atto\,488}}/_{I_{Atto\,680}})$, the simulated curve is consistent with pseudo-second-order sorption kinetics model.[2]

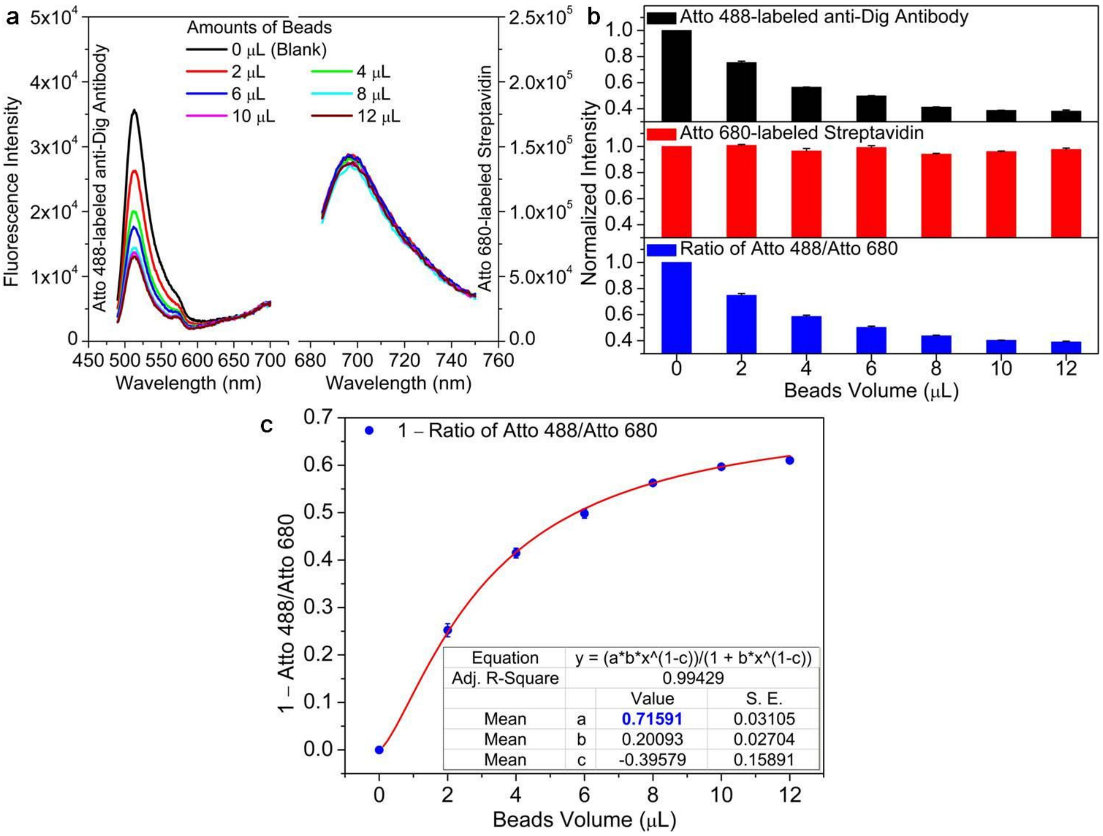

Figure S8. Optimization of the amounts of PS-BSA-Digg beads at the optimal probe concentration in the absence of digoxin. (**a**) Fluorescence emission spectra of Atto 488-labeled anti-Dig antibody (3.33 nM) and Atto 680-labeled streptavidin (60.61 nM) from the

supernatant after being incubated with different amounts of PS-BSA-Digg beads. (**b**) Fluorescence peak intensities of Atto 488 and Atto 680 normalized to the sample in the absence of PS-BSA-Digg beads. The signal ratio of Atto 488/Atto 680 is calculated accordingly. (**c**) The signal on PS-BSA-Digg beads by a simple conversion of $(1 - {}^{I_{Atto\ 488}}/_{I_{Atto\ 680}})$, and the associated Langmuir-Freundlich simulation.

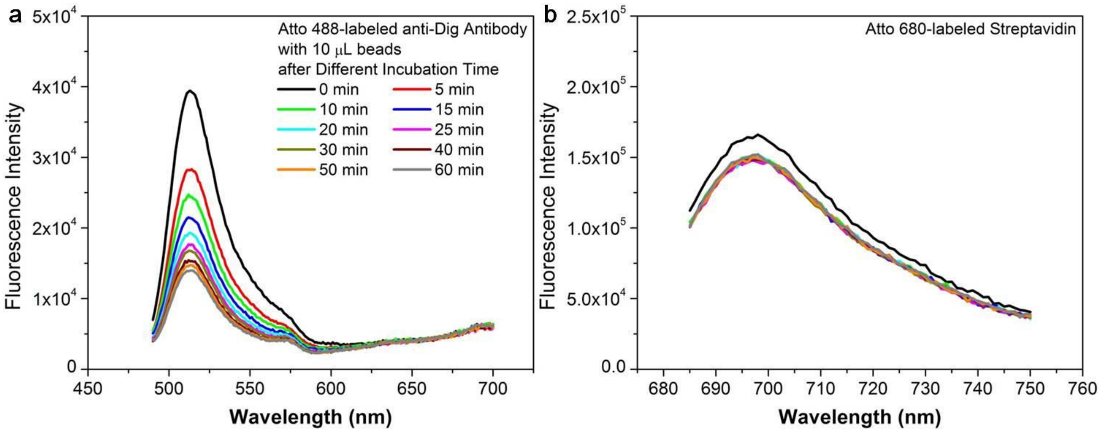

Figure S9. Fluorescence emission spectrum from the supernatant after being incubated with 10 μL PS-BSA-Digg beads for different time: (**a**) Atto 488-labeled anti-Dig antibody (3.33 nM); (**b**) Atto 680-labeled streptavidin (60.61 nM).

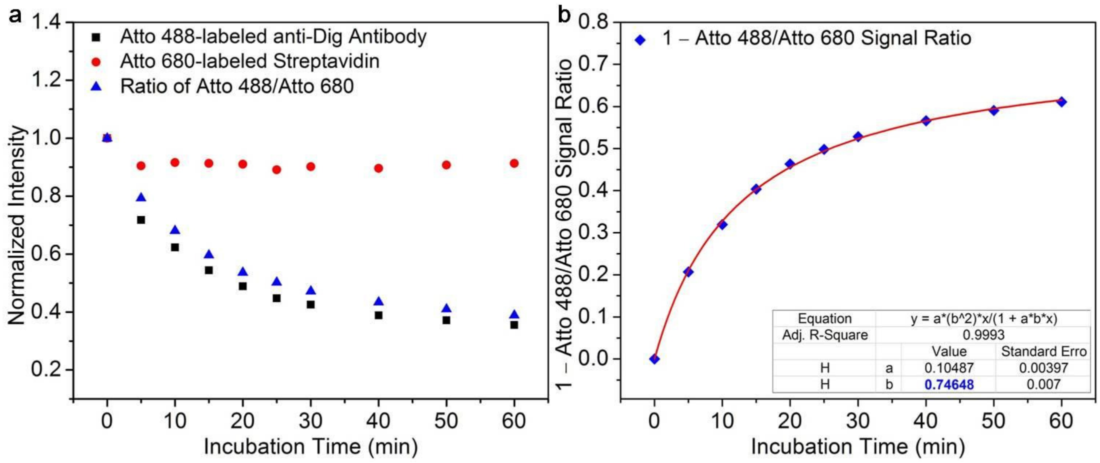

Figure S10. Kinetics study of the immunoadsorption. (**a**) Plot of normalized fluorescence emission intensities from Atto 488-labeled anti-Dig antibody (3.33 nM) and Atto 680-labeled streptavidin (60.61 nM) in the supernatant after being incubated with 10 μL PS-BSA-Digg beads for different time. (**b**) The signal on PS-BSA-Digg beads by a simple conversion of $(1 - {}^{I_{Atto\ 488}}/_{I_{Atto\ 680}})$ and the simulated pseudo-second-order sorption kinetics.

In the following section, we investigate detection of digoxin at different concentrations (**Figure S11**). The samples are first pre-incubated with probes and then PS-BSA-Digg beads are added to capture the excess Atto 488-labeled anti-digoxin antibody. From the signals in the supernatant (**Figure S11a** and **S11b**), the fluorescence intensity from Atto 488-labeled antibody gradually becomes stronger gradually with the increment of digoxin concentration while the fluorescence intensity from Atto 680-labeled streptavidin remains almost the same.

The normalized fluorescence is shown in **Figure S11c** with the corresponding signal ratio at the bottom of **Figure S11c**, which provides a dynamic and reliable response to different concentrations. The present method has a detection range of 0.2-6 nM with a practical detection limit of 0.2 nM (**Figure S11d**), which covers the range of clinical monitoring of digoxin.

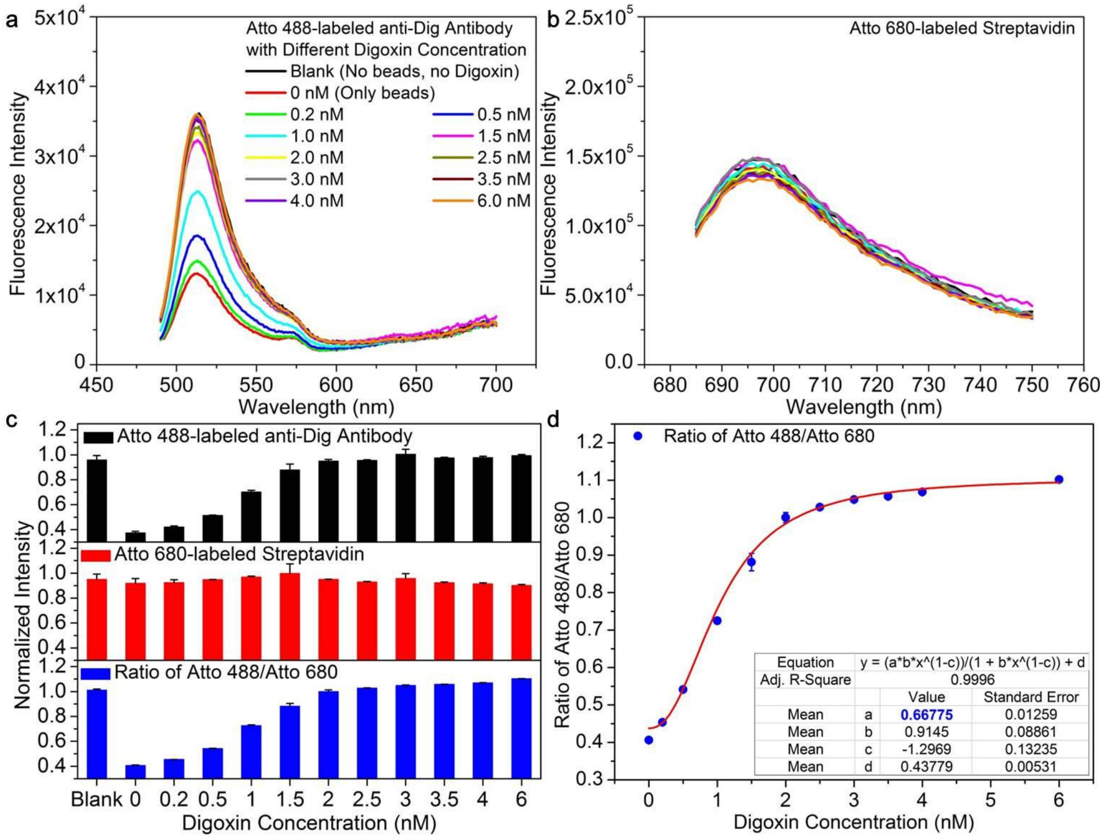

Figure S11. Responses of the assay to digoxin at different concentrations. Fluorescence emission spectra of (**a**) Atto 488-labeled anti-Dig antibody (3.33 nM) and (**b**) Atto 680-labeled streptavidin (60.61 nM) from the supernatant after being incubated with different concentrations of digoxin and then incubated with PS-BSA-Digg beads (10 µL) followed by removal of the beads. (**c**) Normalized fluorescence intensities of Atto 488 and Atto 680. The signal ratio of Atto 488/Atto 680 is calculated accordingly. (**d**) Concentration regression curve (Langmuir-Freundlich simulation in inset). The concentrations given in c and d correspond to the digoxin concentrations in 200 µL buffer. Plotted values are mean values with standard deviations compared to the mean value (N=3).

Section S4 Consistence between simulations and experimental results

It should be noted that in all the simulations above (**Figure S8c**, **S10b**, and **S11d**), each has a parameter (0.71591, 0.74543, and 0.66775, respectively) in the corresponding function (indicated in bold blue font in respective insert, see Table S2 for detailed information of functions). They all refer to a parameter of about 0.7, which corresponds to the maximum sorption on modified beads. This also means that the minimum background in the supernatant should be about 0.3. However, the lowest background obtained is about 0.4 (0.39, 0.39, and 0.40 in **Figure S8**, **S10**, and **S11**, respectively), which means the signal in solution without any active Atto 488-labeled antibody. As indicated by the kinetics study in **Figure S10b**, where the immunoadsorption deteriorates in the later stage before reaching the full balance, it might take a little longer time to finally equilibrate. Therefore, the samples are incubated overnight and then the fluorescence signals from the supernatant are measured and processed to further calculate the signal ratio. The result is consistent with all the above simulations (**Figure S12**) and further confirms the reliability of the present method as well as the two signal-mode strategy.

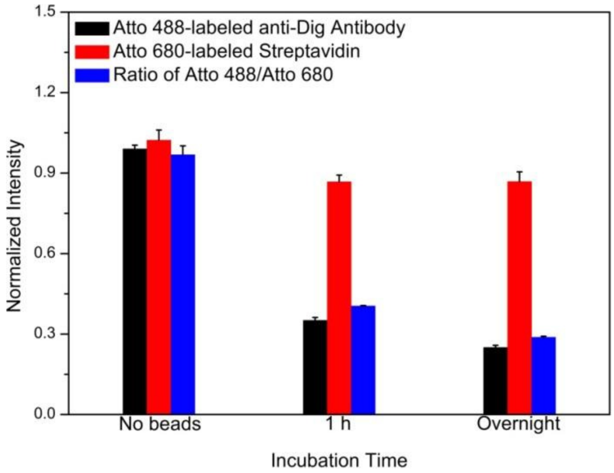

Figure S12. Normalized fluorescence intensity of Atto 488-labeled anti-Dig antibody (3.33 nM) and Atto 680-labeled streptavidin (60.61 nM) from blank solution with no PS-BSA-Digg beads or supernant by separating the beads after different incubation time (10 µL PS-BSA-Digg beads are used).

Section S5 Investigation of specificity and assay precision

The specificity of the detection method is further investigated by treating the system with a series of compounds. These compounds either have similar or related structure, or are pharmaceutically relevant. The responses of the system are processed from samples spiked with individual substance (**Figure S13** and **S14a**).

The Concentration Difference (CD) is calculated for each molecule according to Eq S2, and the Cross Reactivity (CR, given by Eq S3) is calculated by deriving a ratio between Concentration Difference (CD) to the tested compound concentration. From the results shown in **Figure S14c** and **S14d**, it appears that the interference of all the selected compounds on the detection system is minimal.

CD = Measured Digoxin Concentration of sample spiked with test compound (C_{Test}) – Digoxin Concentration of control (C_{Ctrol}) (S2)

$$CR = \frac{Concentration\ Difference}{Test\ Compound\ Concentration} \qquad (S3)$$

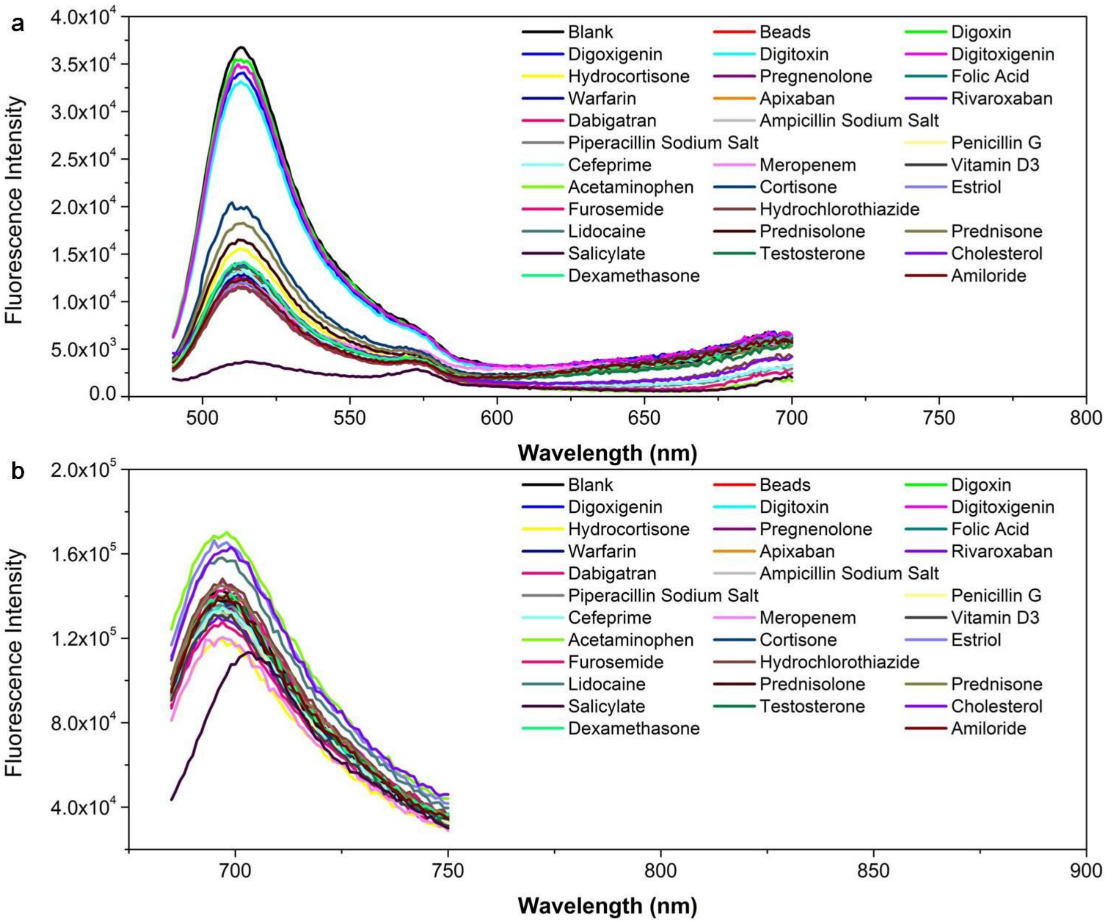

Figure S13. Fluorescence emission spectrum of (A) Atto 488-labeled anti-Dig antibody (3.33 nM) and (B) Atto 680-labeled streptavidin (60.61 nM) from the detection system in the presence of different small molecules at fixed concentrations indicated in **Figure S14**.

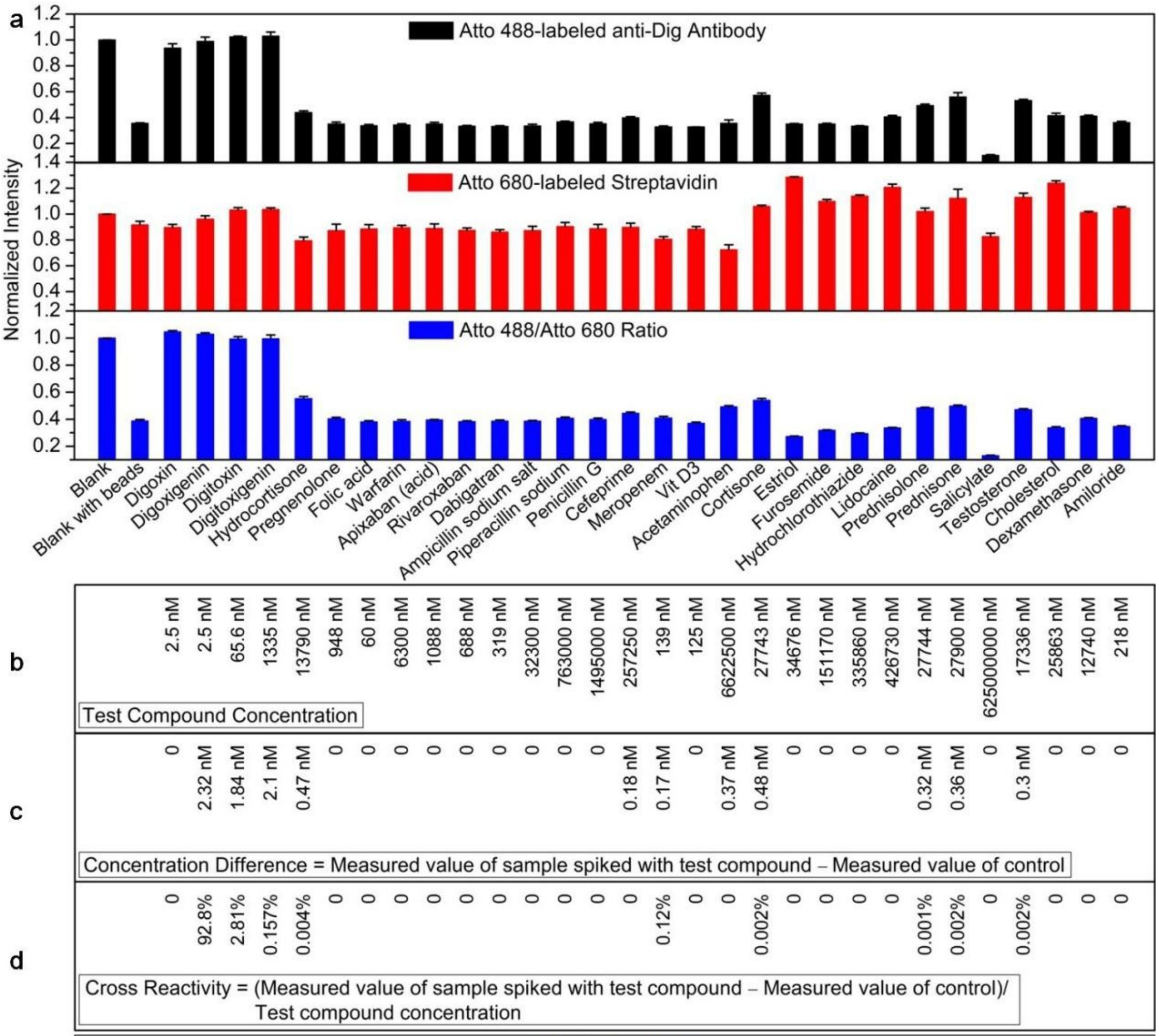

Assay Precision					
	**Number**	**Spiked (nM)**	**Mean (nM)**	**S. D.**	**C. V.**
Low	10	0.6	0.563	0.02	3%
Middle	10	1.6	1.51	0.1	7%
High	10	2.6	2.42	0.13	6%

Figure S14. Specificity and assay precision. (**a**) Normalized fluorescence emission intensity of Atto 488-labeled anti-Dig antibody (3.33 nM) and Atto 680-labeled streptavidin (60.61 nM) from the detection system in response to different molecules of the same kind of drug or with analogous structure, and the corresponding signal ratios. (**b**) The concentration of each investigated small molecules in the detection system. (**c**) The calculated Concentration Difference for each molecule. (**d**) Calculated Cross Reactivity for each molecule. (**e**) Assay precision investigation at three different levels of concentration. Plotted values are mean values with standard deviations compared to the mean value (N=3).

As expected, the response of the system to digoxin and digoxigenin is almost the same because they share main structure which is recognized by the labeled antibody. Although digitoxin and digitoxigenin give high fluorescence responses, due to their structural relation to digoxin, the corresponding Cross Reactivity is low at administered concentrations. All the results in **Figure S14a-d** indicate high specificity of the developed method for digoxin detection. The assay precision is further investigated by analyses on three different levels of

pool control samples. Table in **Figure S14e** shows the number of test times, spiked concentration, mean values determined, standard deviation (S.D.) and coefficient of variation (C.V.) for each of these control samples. The method has an assay precision of < 10% C.V.

Section S6 Recycle the modified beads

To study recycling of the PS-BSA-Digg beads, the collected beads used in the prior experiments are incubated with a commercial dissociation buffer that disrupts the binding between anti-Dig and digoxin/digoxigenin (**Figure S15**, **S16**, and **S17**). The dissociation of anti-Dig from the PS-BSA-Digg beads is very fast. After 1 minute the dissociation of the anti-Dig antibody from the beads is almost complete and it takes only 10-15 min to reach the same background as fresh PS-BSA-Digg beads. To assess whether the PS-BSA-Digg beads conserve their activity after the dissociation procedure, the recycled beads were applied to the detection system again. In **Figure S15b**, **S15c**, and **S17b**, it appears that the recycled beads treated with dissociation buffer from 15 seconds to 3.5 h are good as fresh beads. Longer dissociation times at 1-3h do not destroy the modified beads or influence the activity. In the experiments shown in **Figure S16** and **S17c**, the beads are recycled 15 times. Furthermore, all the following experiments were done by recycling the beads up to 50 times. The error bars from the signal ratios only increases slightly after extended recycling the beads, because the recycling process involves many rounds of washing and centrifugation, which may cause some loss of beads in the process.

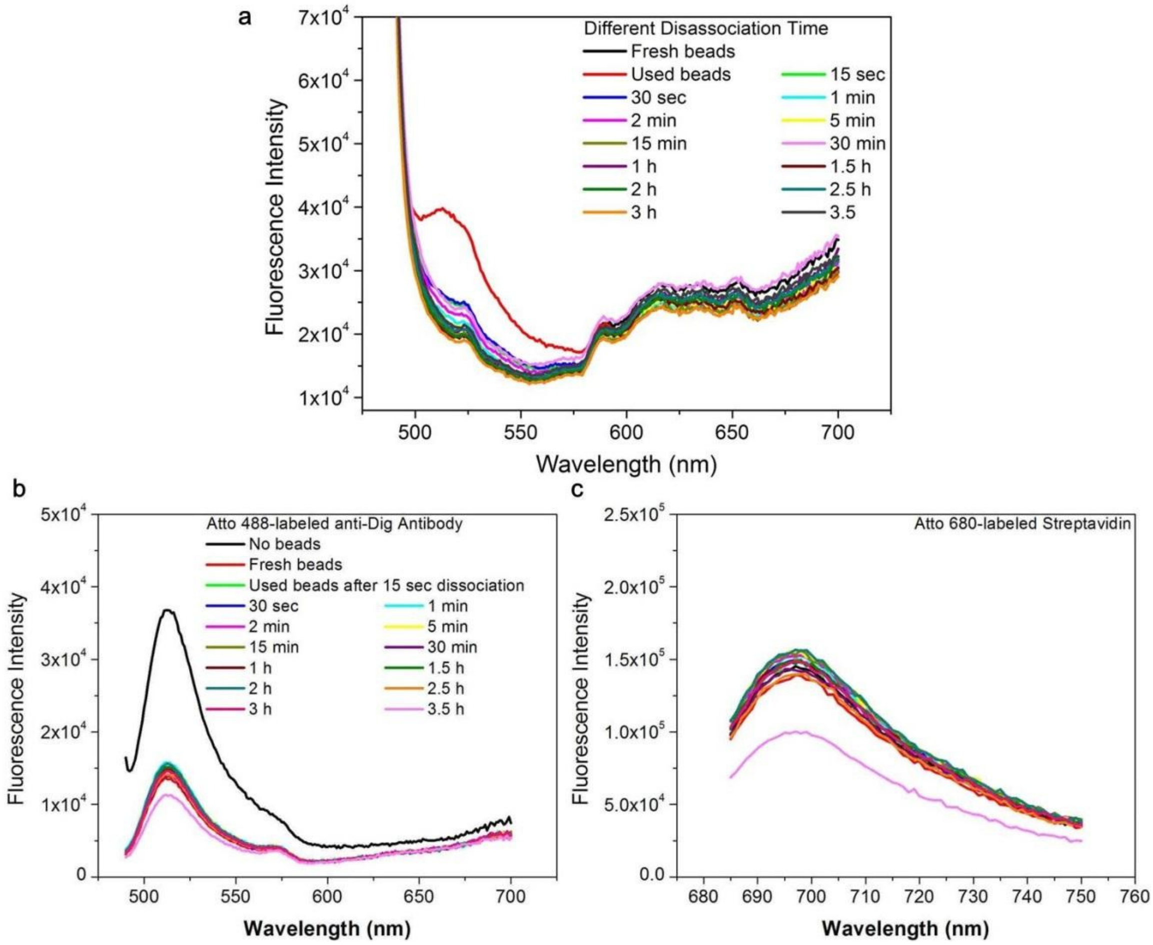

Figure S15. (**a**) Fluorescence emission spectrum from a suspension of recycled PS-BSA-Digg beads with adsorption of Atto 488-labeled anti-Dig antibody after being incubated with dissociation buffer for different time. (**b**) and (**c**) are fluorescence emission spectrum of Atto 488-labeled anti-Dig antibody (3.33 nM) and Atto 680-labeled streptavidin (60.61 nM), respectively, with the use of recycled beads after different incubation time in dissociation buffer.

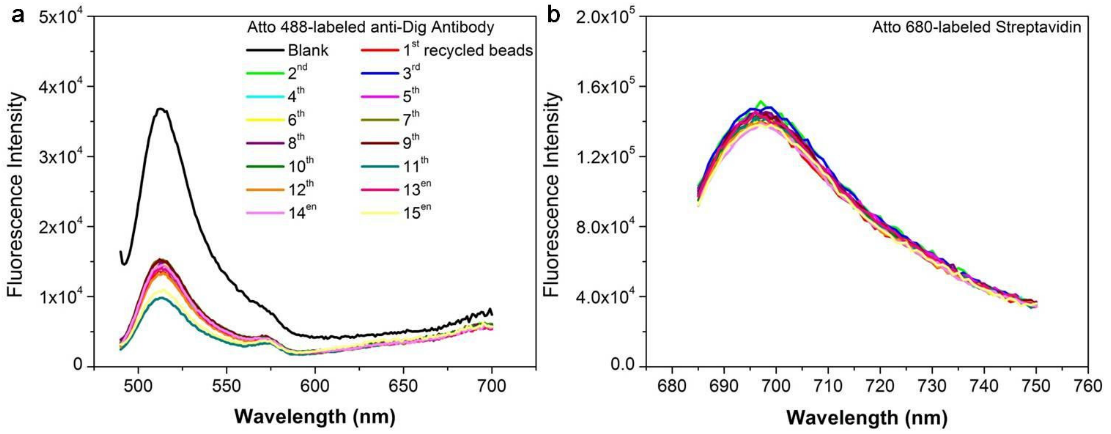

Figure S16. Fluorescence emission spectrum of (**a**) Atto 488-labeled anti-Dig antibody (3.33 nM) and (**b**) Atto 680-labeled streptavidin (60.61 nM), respectively, from the supernatant after centrifugation. PS-BSA-Digg beads are recycled for different times.

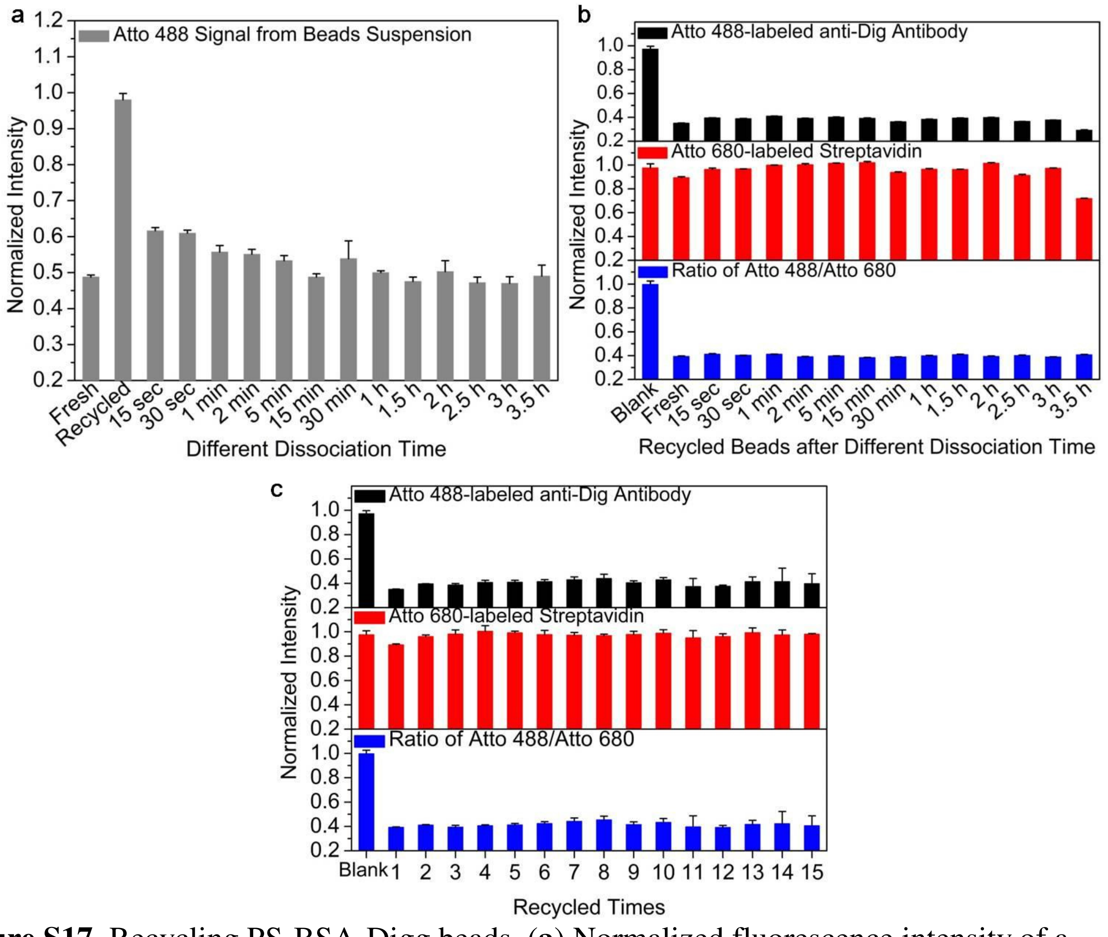

Figure S17. Recycling PS-BSA-Digg beads. (**a**) Normalized fluorescence intensity of a suspension of used PS-BSA-Digg beads treated with dissociation buffer for different time. (**b**) Normalized fluorescence emission intensity of Atto 488-labeled anti-Dig antibody (3.33 nM) and Atto 680-labeled streptavidin (60.61 nM), and the corresponding signal ratio with the use of PS-BSA-Digg beads recycled by different treating time from (**a**). (**c**) Test recycled times with the use of recycled beads from (**a**) with dissociation time of 10 min.

The performance of recycled beads in the detection of digoxin at different concentrations is further investigated (**Figure S18**, **S19**, **S20**, **S21b**, and **S22**). Although the concentration

regression curve is a little different from that with fresh beads (**Figure** S**21a** and S**21e**), the difference is not significant and it might be ascribed to the following reasons: i) there is a slight change of the modified beads' surface because the linked BSA is somewhat denatured in the process of recycling, ii) There may be trace of chemical residues from the immobilization of digoxigenin in the suspension of fresh beads, but they are removed in the process of recycling.

Section S7 Investigation of the influence of plasma on the detection system

Detection of digoxin at different concentrations is performed with detection buffer containing 5% and 15% plasma, respectively (**Figure S21c** and **S21d**). From the comparison of the plotted concentration regression curves deriving from detection in buffer, 5% plasma, and 15% plasma (**Figure S21e**), it is evident that the influence of plasma on the detection is minimal, which confirms the high robustness of the assay. In these experiments, the beads are also recycled. The responses of the system to 1.5 nM digoxin in 25% and 50% plasma are also investigated (**Figure S21f**). A concentration of digoxin of 1.5 nM is chosen for spiking because it is at the most sensitive area in the detection range. The responses of the detection system to samples spiked with 1.5 nM digoxin in detection buffer containing 0%, 5%, 15%, 25%, and 50% plasma are compared, and P values are calculated between them. All P values are above 0.05 (**Figure S22e** insert), which means that there are no significant differences between them and it further confirms the high robustness of the method.

We also tested detection in pure plasma, however, it becomes difficult to separate the modified beads by centrifugation due to the high viscosity of pure plasma. Therefore, the assay works better with diluted plasma. In that regard, another advantage of the present two signal-mode strategy is that the final signal ratio should in principle be independent of the dissolution. To confirm this, fluorescence intensities are measured from differently diluted detection system and the final signal ratios are determined (**Figure S23**). Both fluorescence signals from Atto 488-labeled anti-Dig antibody and Atto 680-labeled streptavidin decrease gradually with the increment of dilution. However, the signal intensity from Atto 680-labeled streptavidin decreases a little more at higher dilutions, which may be ascribed to the fact that the adhesive property of streptavidin could induce more interference at more diluted concentrations. Therefore, the final signal ratio increases slightly with the increment of dilution, but the variation extent is below 20% even when the detection system is diluted 1:9. As the P value indicates, there is no significant difference when it is diluted 1:2. The digoxin concentration in diluted plasma can be determined according to the above-obtained concentration regression curve (**Figure S21b**), which can be converted to the digoxin concentration in the original pure plasma through a simple calculation (Protocol 1b in Methods). Alternatively, to read out digoxin concentration directly from the original plasma sample, we also made a concentration regression curve by taking the dilution into account (**Figure S24b**). Here, pure plasma is diluted by buffer in a ration of 1:2 (**Protocol 1a** in Methods), and the probe concentrations for the detection system are also diluted 1:2 and the results are shown in **Figure S24a** and **S24b** (see simulations in **Figure S22**). The concentration regression curves obtained in buffer and in diluted plasma are almost identical (**Figure S24c**). According to **Protocol 1a** and the concentration regression curve in **Figure S24c**, digoxin concentration in pure plasma can be determined directly.

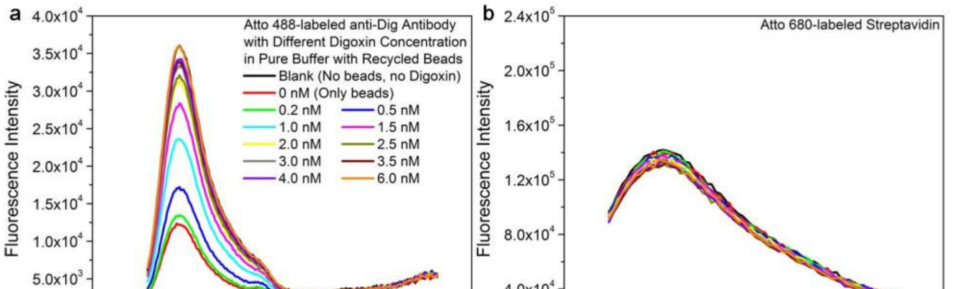

Figure S18. Fluorescence emission spectrum of (**a**) Atto 488-labeled anti-Dig antibody (3.33 nM) and (**b**) Atto 680-labeled streptavidin (60.61 nM), respectively, in the presence of

digoxin at different concentrations in the detection buffer after incubated with recycled PS-BSA-Digg beads and centrifugation.

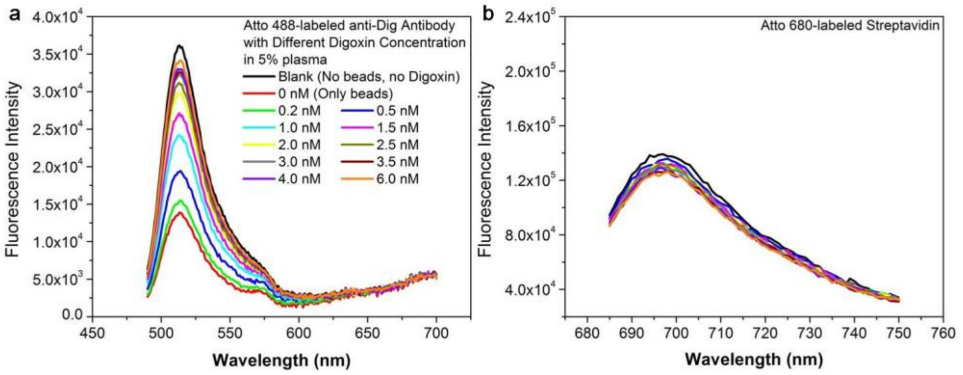

Figure S19. Fluorescence emission spectrum of (**a**) Atto 488-labeled anti-Dig antibody (3.33 nM) and (**b**) Atto 680-labeled streptavidin (60.61 nM), respectively, in the presence of digoxin at different concentrations in 5% plasma after incubation with recycled PS-BSA-Digg beads and centrifugation.

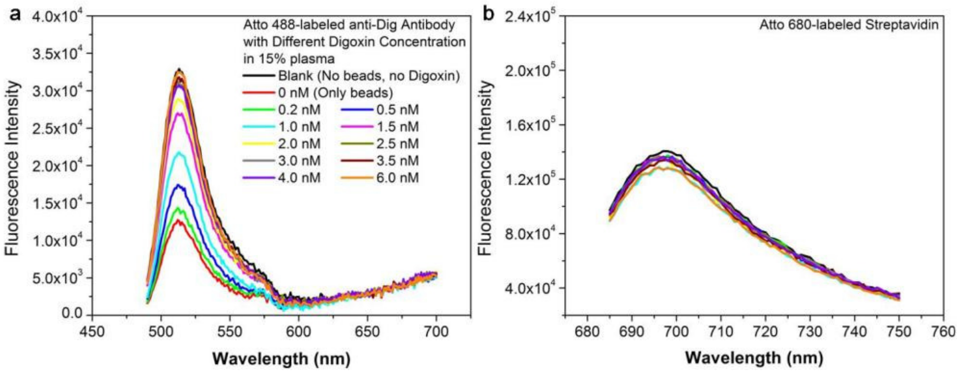

Figure S20. Fluorescence emission spectrum of (**a**) Atto 488-labeled anti-Dig antibody (3.33 nM) and (**b**) Atto 680-labeled streptavidin (60.61 nM), respectively, in the presence of digoxin at different concentrations in 15% plasma after incubation with recycled PS-BSA-Digg beads and centrifugation.

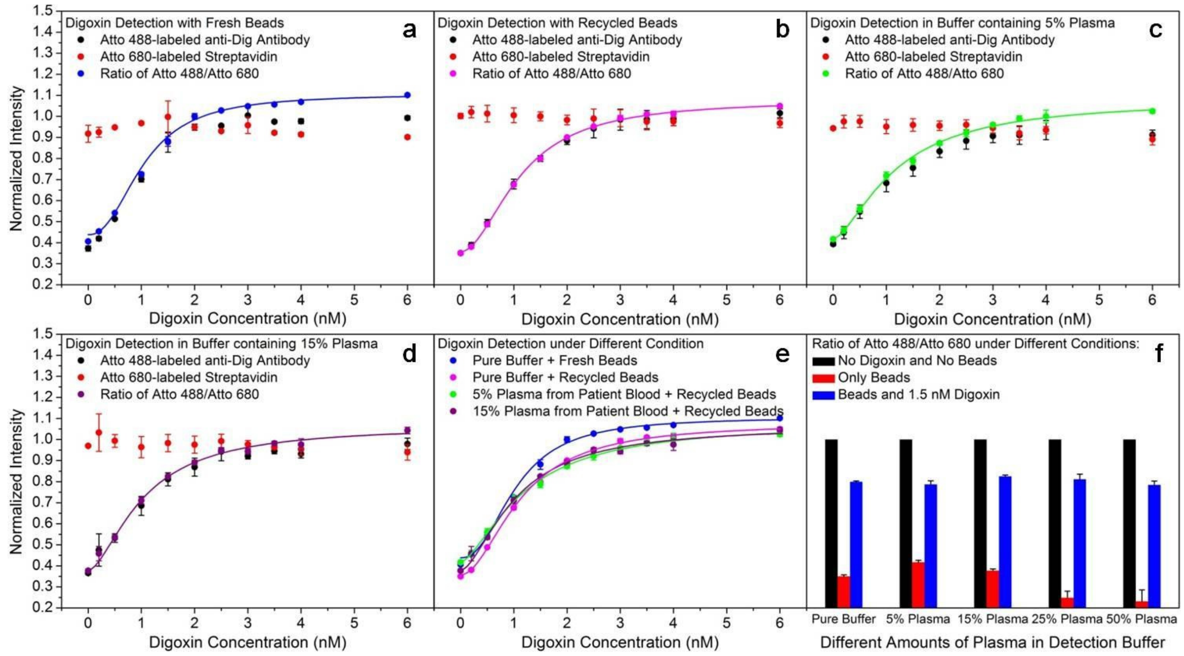

Figure S21. Investigation of the recycling of the PS-BSA-Digg beads and the influence of plasma on the model detection system. Plots of normalized fluorescence intensity of Atto 488-labeled anti-Dig antibody (3.33 nM) and Atto 680-labeled streptavidin (60.61 nM), and the corresponding signal ratio in digoxin detection under different conditions: (**a**) Fresh PS-BSA-Digg beads + detection buffer; (**b**) Recycled PS-BSA-Digg beads + detection buffer; (c) Recycled PS-BSA-Digg beads + detection buffer containing 5% plasma; (**d**) Recycled PS-BSA-Digg beads + detection buffer containing 15% plasma; (**e**) Comparison of concentration regression curves from (a), (b), (c), and (d). (**f**) Comparison of the system responses to samples spiked with 1.5 nM digoxin in detection buffer containing 0%, 5%, 15%, 25%, and 50% plasma, respectively. Concentrations on the x-axes are the concentration of digoxin in final solution. All concentration regression curves are simulated based on Langmuir-Freundlich equation (**Figure S22**). Plotted values are mean values with standard deviations compared to the mean value (N=3).

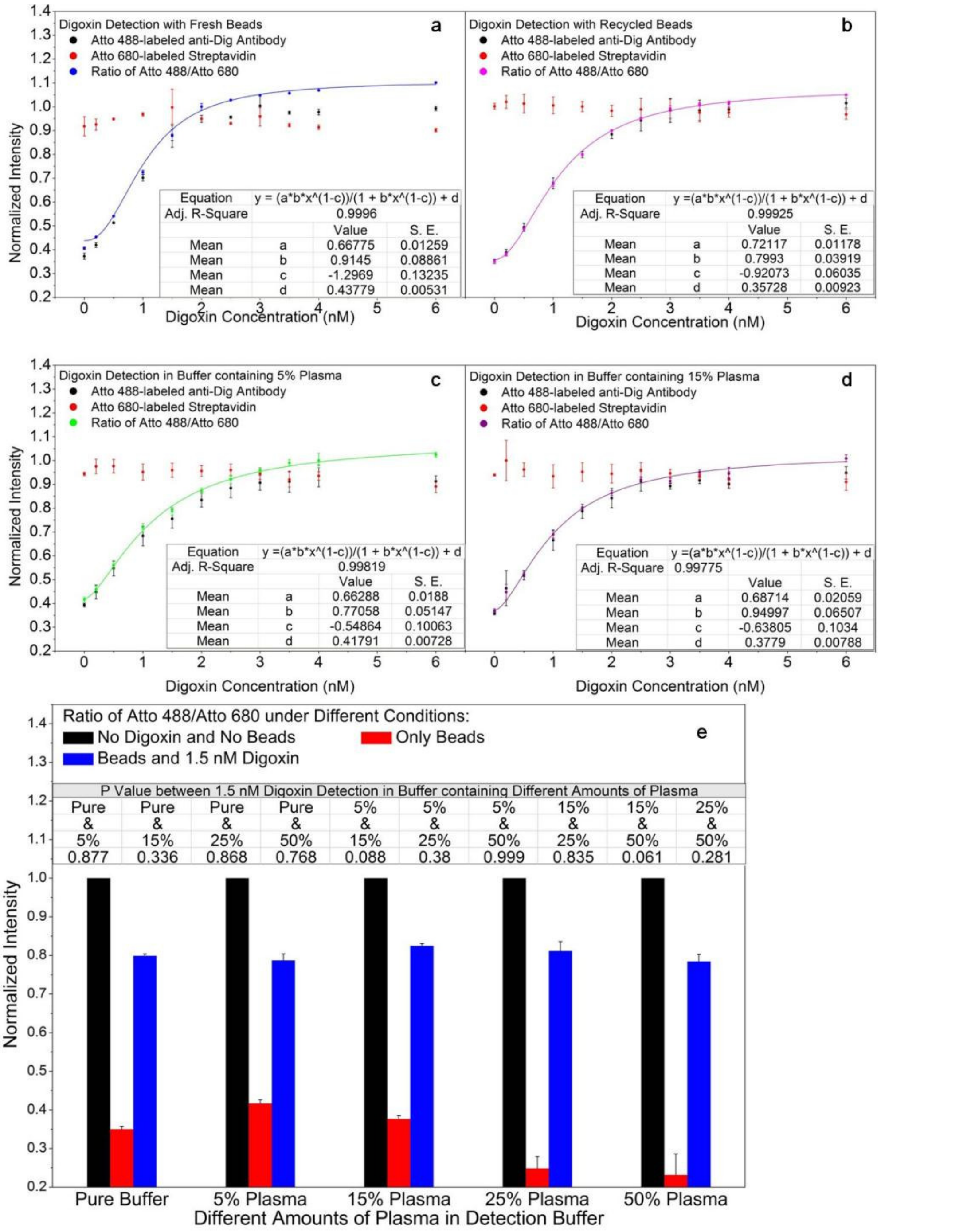

Figure S22. Investigation the influence of plasma on the model detection system. Plots of normalized fluorescence intensity of Atto 488-labeled anti-Dig antibody (3.33 nM) and Atto 680-labeled streptavidin (60.61 nM), and the corresponding signal ratio from digoxin detection under different conditions: (**a**) Fresh PS-BSA-Digg beads + detection buffer; (**b**) Recycled PS-BSA-Digg beads + detection buffer; (**c**) Recycled PS-BSA-Digg beads + detection buffer containing 5% plasma; (**d**) Recycled PS-BSA-Digg beads + detection buffer containing 15% plasma. (**e**) Comparison of the system responses to samples spiked with 1.5 nM digoxin in detection buffer containing 0%, 5%, 15%, 25%, and 50% plasma, respectively (inserted table shows calculated P value between them). All concentration regression curves are simulated based on Langmuir-Freundlich equation (plus a constant parameter d to the function shown in the insert). Plotted values are mean values with standard deviations compared to the mean value (N=3).

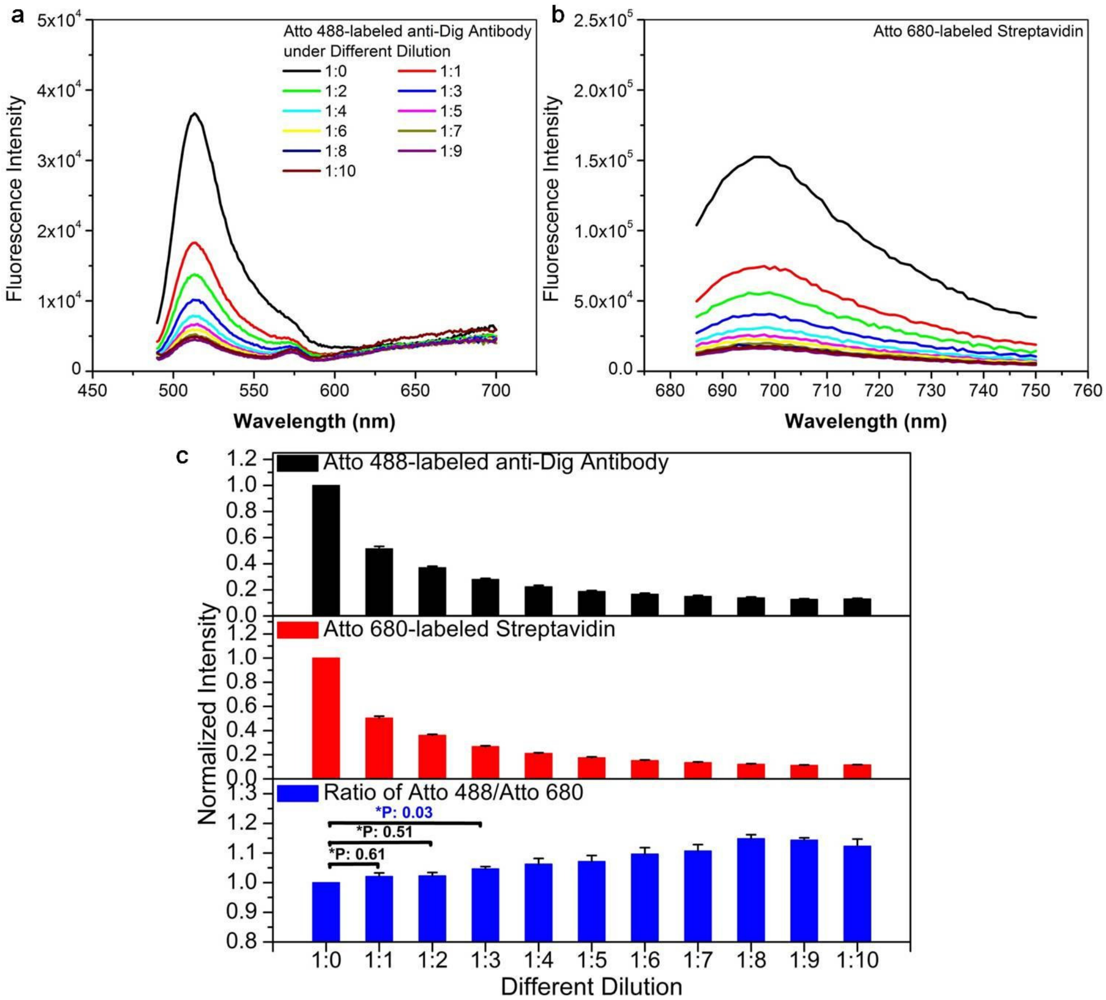

Figure S23. Fluorescence emission spectrum of (**a**) Atto 488-labeled anti-Dig antibody (3.33 nM) and (**b**) Atto 680-labeled streptavidin (60.61 nM), respectively, upon different dilutions with detection buffer. (**c**) Normalized fluorescence intensity of (**a**) and (**b**), and calculation of the signal ratios of Atto 488/Atto 680. P values were calculated to compare signal ratios between three different dilutions.

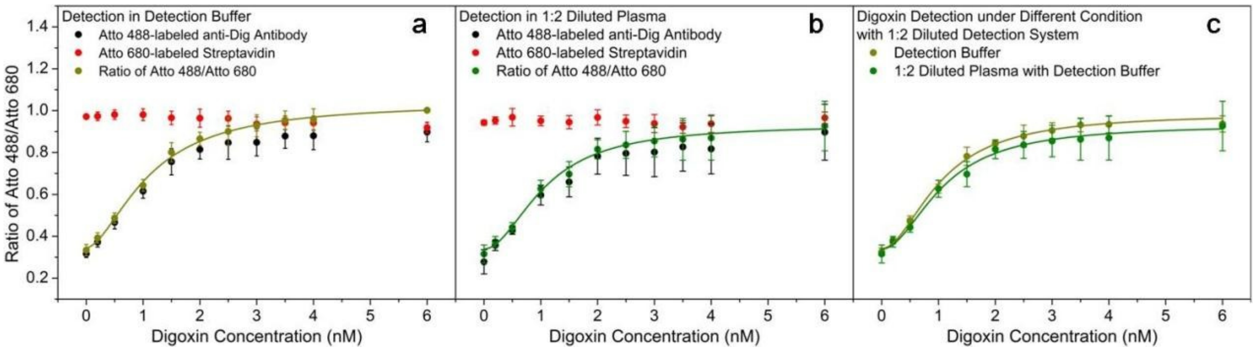

Figure S24. Digoxin detection in plasma diluted 1:2. Plots of normalized fluorescence intensity of Atto 488-labeled anti-Dig antibody (1.11 nM) and Atto 680-labeled streptavidin (20.20 nM) (1:2 diluted probe system), and the corresponding signal ratio in digoxin detection under different conditions: (**a**) Detection buffer; (**b**) 1:2 Diluted plasma with detection buffer. (**c**) Comparison of plotted concentration regression curves from (a) and (b). The concentrations given in all graphs are before dilution. All used PS-BSA-Digg beads are recycled beads. Both concentration regression curves are simulated based on Langmuir-Freundlich equation (**Figure S25**). Plotted values are mean values with standard deviations compared to the mean value (N=3).

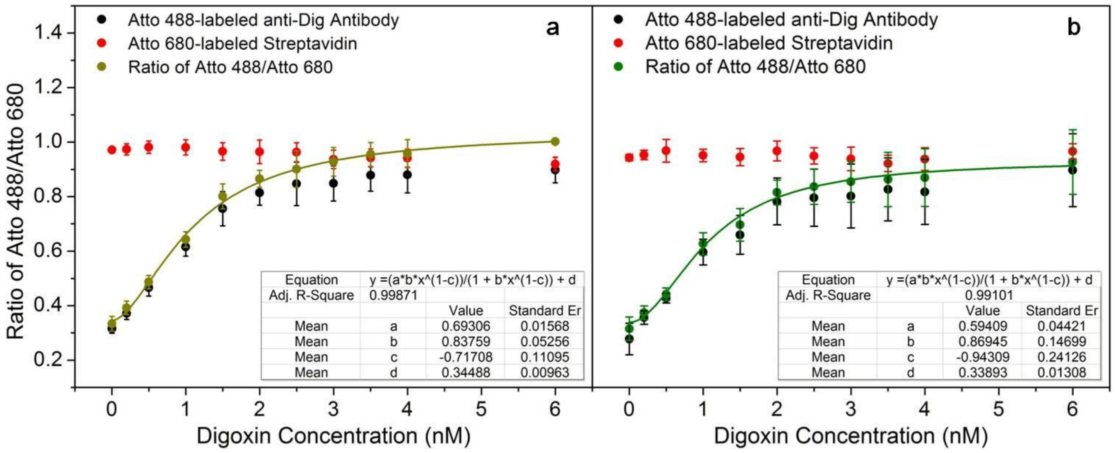

Figure S25. Digoxin detection in plasma diluted 1:2. Plots of normalized fluorescence intensity of Atto 488-labeled anti-Dig antibody (1.11 nM) and Atto 680-labeled streptavidin (20.20 nM) (1:2 diluted probe system), and the corresponding signal ratio in digoxin detection under different conditions: (**a**) Detection buffer; (**b**) 1:2 Diluted plasma with detection buffer. The concentrations given in all graphs are before dilution. All used PS-BSA-Digg beads are recycled beads.Both concentration regression curves are simulated based on Langmuir-Freundlich equation (plus a constant parameter d to the function shown in the insert). Plotted values are mean values with standard deviations compared to the mean value (N=3).

Section S8 G-Chip Operation

The G-Chip mold is designed using AutoCAD (Autodesk) and fabricated by photolithography molding techniques. The G-Chip is composed of molded polydimethylsiloxane (PDMS), and each chip has two wells for inlet and outlet, respectively. The depth of the flow channel in the G-Chip is about 40 µm (**Figure S26**). The chip is designed with multiple layers of filters in the form of pillars that are embedded in the flow channel to retain the 20 µm PS-BSA-Digg beads.

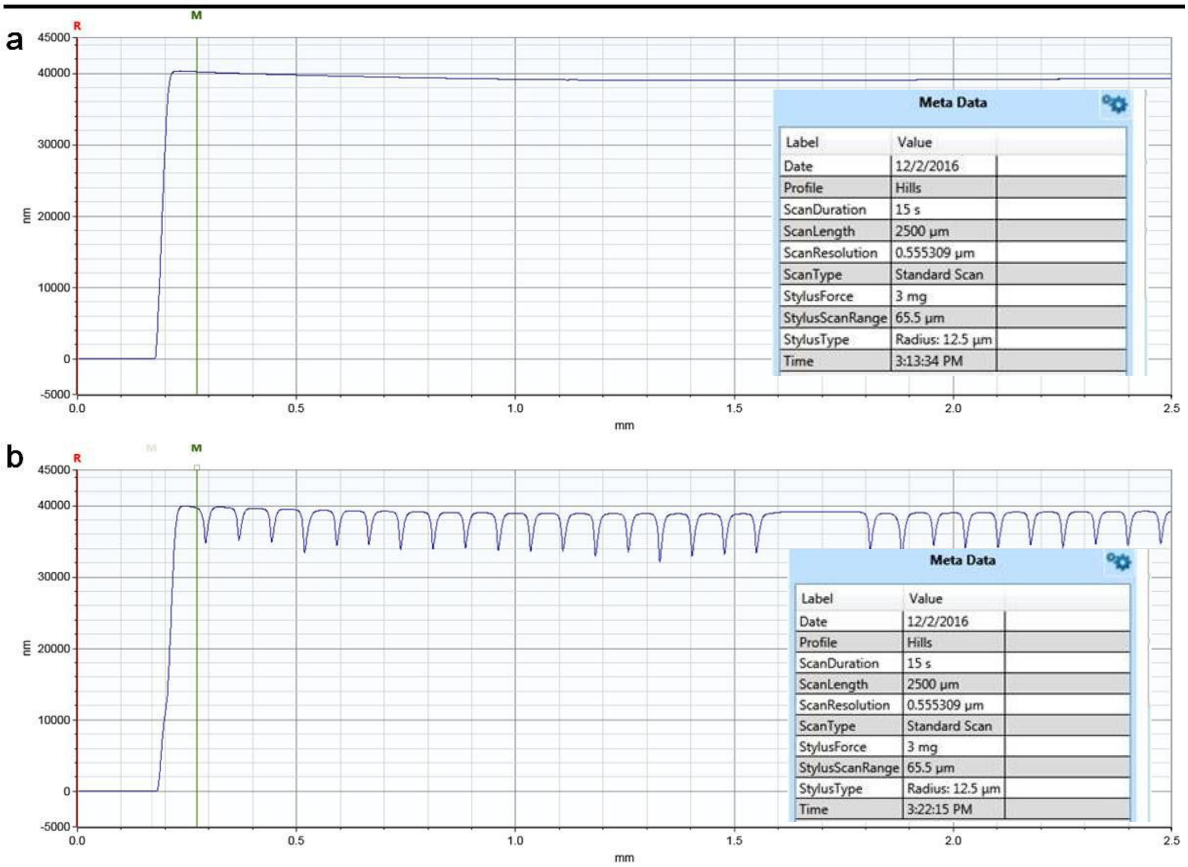

Figure S26. Depth of the mold layer on the wafer scanned across (**a**) Blank areas with no filter pillars; (**b**) areas with filter pillars. The depth of G-Chip flow channel is about 40 µm.

In the operation of the chip, it is first loaded with the PS-BSA-Digg beads that are retained in the filter pillars of the chip. When the fluid flows through chip, the anti-Dig antibodies that are not occupied by binding to digoxin in pre-incubation will be captured on the PS-BSA-Digg beads retained in the chip (see **Figure S27a** for results with unoptimized conditions). Furthermore, it turned out that pre-incubation could be avoided, as similar results were obtained when the plasma sample and the protein probes were loaded into the inlet together (**Figure S27b**).

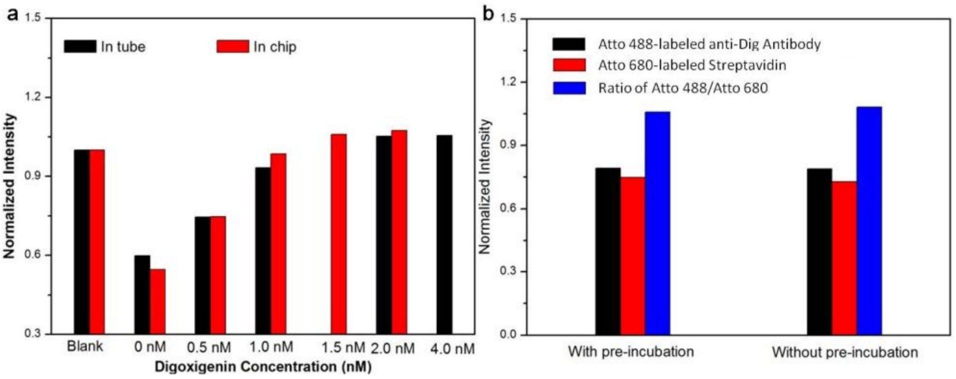

Figure S27. (**a**) Comparison of signal ratios of the detection system in response to digoxin at different concentrations between results from tube and G-Chip with unoptimized conditions (two columns are missed due to different concentration series). (**b**) Comparison of the un-optimized detection system in response to 1 nM digoxin with and without pre-incubation before loading to prepared G-Chip.

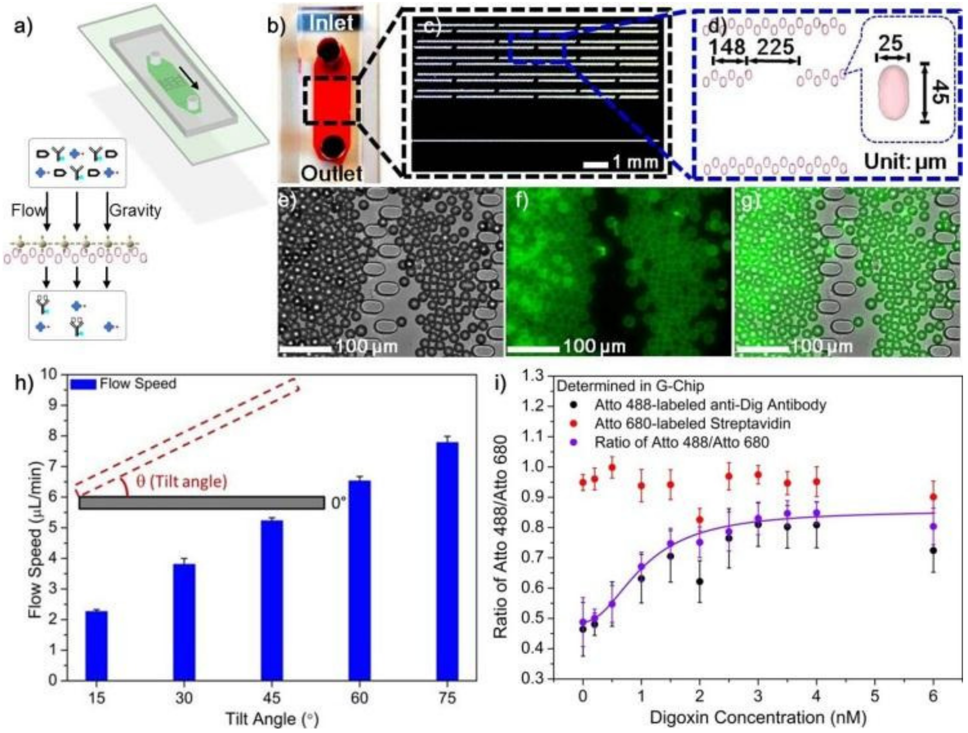

Figure S28. Illustration and photos of the G-Chip and comparison of digoxin detection in plasma using the G-Chip and a tube with only 10 µL plasma. (**a**) Schematic view of the designed G-Chip. (**b**) A typical optical image of the fabricated chip. (**c**) Image of filter pillars in the middle of fluid channel. (**d**) Microscopy of the filter design (insert shows the dimensions of each pillar from the filter). (**e**) Bright-field and (**f**) fluorescence image of the chip filters loaded with 20 µm PS-BSA-Digg beads with adsorption of Atto 488-labeled anti-Dig antibody. (**g**) The merged image of (**e**) and (**f**). (**h**) The flow speed through the channel of the G-Chip at different tilt angles. (**i**) Plots of normalized fluorescence intensity of Atto 488-labeled anti-Dig antibody (0.42 nM) and Atto 680-labeled streptavidin (7.58 nM) (1:7 diluted probe system), and the corresponding signal ratio at different digoxin concentrations in 1:7 diluted plasma from G-Chip. The concentrations given in **i** are of digoxin in plasma before dilution. All used PS-BSA-Digg beads are recycled beads. Plotted values are mean values with standard deviations compared to the mean value (N=3).

For detection of digoxin in the G-Chip (**Figure S28**), only 10 µL blood plasma is required, which is mixed with the labeled anti-Dig antibody and streptavidin probes in 70 µL in the inlet. As the volume of 10 µL is only 1/20 of the total volume of one sample in the previous detection with the use of detection buffer, accordingly, we applied a 1/20 protocol for the detection system in this case (see Protocol 1c for tube and **Protocol 2a** for the G-Chip in Methods). After loading of the mixture into the chip inlet by a pipette, the chip is tilted 30° from the horizontal orientation. After the solution has passed through the chip and the non-occupied antibodies have been retained on the PS-BSA-Digg beads, the solution is collected at the outlet of the chip and the fluorescence of the Atto 488-labeled anti-Dig antibody and Atto 680-labeled streptavidin is monitored to determine the ratio and in turn the concentration of digoxin (**Figure S29a**). For comparison, the results obtained from monitoring digoxin in a tube are shown in **Figure S29b** and it is clear that the response and the error bars are quite similar (**Figure S29c**, simulations in **Figure S30**). The slight differences may be ascribed to the following reasons: i) some degree of photobleaching during the G-Chip handling, ii) some degree of unspecific binding of antibody to the PDMS surface although it is pre-treated with detection buffer.

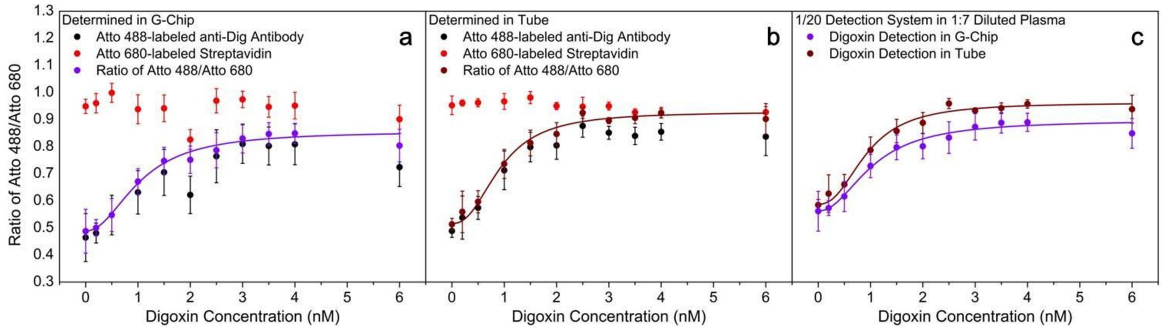

Figure S29. Comparison of digoxin detection in plasma using the G-Chip and a tube with only 10 µL plasma. Plots of normalized fluorescence intensity of Atto 488-labeled anti-Dig antibody (0.42 nM) and Atto 680-labeled streptavidin (7.58 nM) (1:7 diluted probe system), and the corresponding signal ratio at different digoxin concentrations in 1:7 diluted plasma from: (**a**) G-Chip; (**b**) Tube. (**c**) Comparison of plotted concentration regression curves from (a) and (b). The concentrations given in **a-c** are of digoxin in plasma before dilution. All used PS-BSA-Digg beads are recycled beads. Both concentration regression curves are simulated based on Langmuir-Freundlich equation (**Figure S30**). Plotted values are mean values with standard deviations compared to the mean value (N=3).

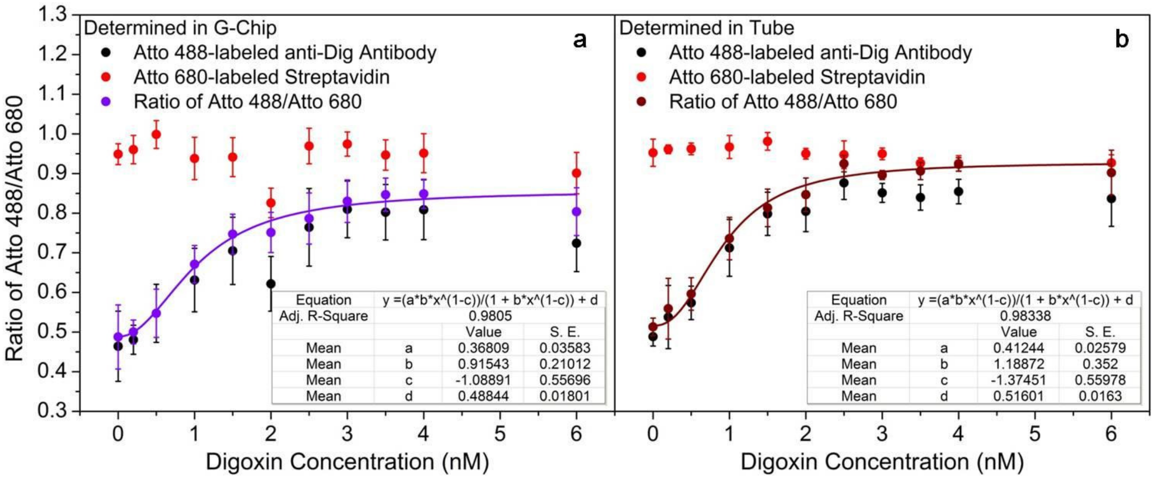

Figure S30. Plots of normalized fluorescence intensity of Atto 488-labeled anti-Dig antibody (0.42 nM) and Atto 680-labeled streptavidin (7.58 nM) (1:7 diluted probe system), and the corresponding signal ratio at different digoxin concentrations in 1:7 diluted plasma from: (**a**) G-Chip; (**b**) Tube. The concentrations given are of digoxin in plasma before dilution. All used PS-BSA-Digg beads are recycled beads. Both concentration regression curves are simulated based on Langmuir-Freundlich equation (plus a constant parameter d to the function shown in the insert). Plotted values are mean values with standard deviations compared to the mean value (N=3).

It only lasts about 20 min to collect about 60 µL liquid from the outlet well. It is fast, and it could be reduced to 10 min if less liquid is required for the fluorescence measurement. It should be mentioned that the G-Chip with the captured PS-BSA-Digg beads are continuously recycled by applying the dissociation buffer directly to the chip, thereby liberating the antibodies bound to the PS-BSA-Digg beads captured in the chip (see **Protocol to recycle the G-Chip** in Methods). Recycled G-Chips have been applied to generate the concentrations regression curve in **Figure S29a** as well as all the following experiments. There is no loss of beads or activity when recycling the beads in the chip, since they stay in the filter pillars of the G-Chip. The results show that the performance of recycled chips is highly stable because the captured PS-BSA-Digg beads, as the core materials, do not change. To increase the precision of the detection of digoxin in the G-Chip at low nanomolar concentrations, a four-chip method has been developed.

Table S2. All simulated functions and respective parameter meaning.

<table>
<tr><th>Inserts from</th><th>Equation</th><th>Parameters</th><th>Meaning</th></tr>
<tr><td rowspan="5">Figure S8</td><td rowspan="5">$Y=(a*b*x^{(1-c)})/(1+b*x^{(1-c)})$</td><td>Y</td><td>Signal on beads by $(1-\frac{I_{Atto\ 488}}{I_{Atto\ 680}})$ conversion</td></tr>
<tr><td>x</td><td>Different amount of beads</td></tr>
<tr><td>a</td><td>Maximum adsorbed signal on beads</td></tr>
<tr><td>b</td><td>Constant</td></tr>
<tr><td>c</td><td>Constant</td></tr>
<tr><td rowspan="4">Figure S10</td><td rowspan="4">$Y=(a*b^2*x)/(1+a*b*x)$</td><td>Y</td><td>Signal on beads by $(1-\frac{I_{Atto\ 488}}{I_{Atto\ 680}})$ conversion</td></tr>
<tr><td>x</td><td>Different time</td></tr>
<tr><td>a</td><td>Constant</td></tr>
<tr><td>b</td><td>Maximum adsorbed signal on beads at equilibrium</td></tr>
<tr><td rowspan="6">Figure S11, S22, S25, and S29</td><td rowspan="6">$Y=(a*b*x^{(1-c)})/(1+b*x^{(1-c)})+d$</td><td>Y</td><td>Signal on beads by $(1-\frac{I_{Atto\ 488}}{I_{Atto\ 680}})$ conversion</td></tr>
<tr><td>x</td><td>Different amount of beads</td></tr>
<tr><td>a</td><td>Maximum adsorbed signal on beads</td></tr>
<tr><td>b</td><td>Constant</td></tr>
<tr><td>c</td><td>Constant</td></tr>
<tr><td>d</td><td>Constant</td></tr>
</table>

Table S3. Comparison of the this work with last-5-year literatures and commercial kits.

Literatures							
No.	Year	Detection Method	Detection Range [nM]	LOD (Limit of Detection, [nM])	Method to obtain LOD	Detection Time	Reference
1	2014	Electrochemical	0.64-0.64	0.06	Calculated	65 min	[3]
2	2014	LCMS	0.64-1280.41	0.639	Practical	16 min for only LCMS	[4]
3	2015	Fluorescent	0-500	0.566	Calculated	>30 min	[5]
4	2015	Fluorescent<br>Colorimetric	0-30	0.392<br>0.571	Calculated	30 min	[6]
5	2015	HRPC/MS	0.2-12.2	0.05-2.56	Calculated	17 min for only HRPC	[7]
6	2015	Electrochemical	0.026-0.26	0.013	Calculated	>45 min	[8]
7	2017	Electrochemical	1-30	1	Practical	~40 min	[9]
8	2017	Electrochemical	0.1×10^{-3}-1×10^{3}	0.05×10^{-3}	Calculated	~120 min	[10]
9	2017	Fluorescent	$\sim2\times10^{6}$-6×10^{6}	$\sim800\times10^{3}$	Calculated	>90 min	[11]
10	2017	Fluorescent	$2\text{-}10\times10^{3}$	0.74	Calculated	90 min	[12]
11	2017	SPR	~12.4-321	2.56	Calculated	120 min	[13]
12	2018	Fluorescent	~0-600	60.5	Calculated	<5 min	[14]
13	2018	Fluorescent	$30\text{-}20\times10^{3}$	28	Calculated	~30 min	[15]
14	2018	Fluorescent	10-100	8.2	Calculated	30 min	[16]
15	2018	Electrochemical	1-50	0.07	Calculated		[17]

Commercial Kits for Digoxin Detection					
No.	Name	Detection Range [nM]	LOD (Limit of Detection, [nM])	Assay Time	Company
1	AccuDiag™ Digoxin (Dig) ELISA Kit	0.64-2.56	0.092	~45 min	Diagnostic Automation/Cortez Diagnostics, Inc.
2	AlphaScreen™ Digoxin/Digoxigenin (DIG) Detection Kit	0-3	Not Available	90 min	PerkinElmer Inc.
3	CEDIA® Digoxin II Assay	0.19-5.12	0.19	~20 min	Thermo Fisher Scientific Inc.
4	Cobas® Digoxin	0.19-6.4	0.19	18 min	Roche Diagnostics GmbH
5	DIG AccuBind® Test System	0.64-3.73	0.092	~45 min	Monobind Inc.
6	Digoxin ELISA Kit	0.64-5.12	0.064	60 min	Novatein Biosciences
7	Digoxin II	0.38-5.12	0.38	10~45 min	Abbott
8	DRI® Digoxin Assay	0.64-6.4	0.13	Not Available	Thermo Fisher Scientific
9	*Emit® 2000* Digoxin Assay	0.3-6.4	0.3	Not Available	BECKMAN COULTER Life Sciences
10	Human Digoxin ELISA Kit	0.064-5.12	0.064	>45 min	CREATIVE DIAGNOSTICS

Commercial Kits for Digoxin Detection					
No.	Name	Detection Range [nM]	LOD (Limit of Detection, [nM])	Assay Time	Company
11	*i*Digoxin	0.38-5.12	0.38	Not Reported	Abbott
	This Work	**0.2-6 nM**	**0.2 nM**	**~40 min for tube; <20 min on G-Chip**	

(Clinical requirements: 0.6-2.6 nM)

References

[1] G. Alberti, V. Amendola, M. Pesavento, R. Biesuz, Coord. Chem. Rev. **2012**, 256, 28-45.
[2] S. Azizian, J. Colloid Inferface Sci. **2004**, 276, 47-52.
[3] A. Ahmadi, H. Shirazi, N. Pourbagher, A. Akbarzadeh, K. Omidfar, Mol. Biol. Rep. **2014**, 41, 1659.
[4] H. Yamaguchi, K. Miyamori, T. Sato, J. Ogura, M. Kobayashi, T. Yamada, N. Mano, K. Iseki, J. Chromatogr. B **2014**, 972, 73.
[5] A. S. Emrani, S. M. Taghdisi, N. M. Danesh, S. H. Jalalian, M. Ramezani, K. Abnous, Anal. Methods **2015**, 7, 3814.
[6] A. S. Emrani, N. M. Danesh, P. Lavaee, S. H. Jalalian, M. Ramezani, K. Abnous, S. M. Taghdisi, Anal. Methods **2015**, 7, 3419.
[7] C. Bylda, R. Thiele, U. Kobold, D. A. Volmer, Drug Test. Anal. **2015**, 7, 937.
[8] H. Bagheri, R. P. Talemi, A. Afkhami, Rsc Advances **2015**, 5, 58491.
[9] S. S. Mahshid, F. Ricci, S. O. Kelley, A. Vallee-Belisle, ACS Sens. **2017**, 2, 718.
[10] M. H. Mashhadizadeh, A. Azhdeh, N. Naseri, J. Electroanal. Chem. **2017**, 787, 132.
[11] M. M. Sebaiy, C. L. Colyer, Anal. Lett. **2017**, 50, 2530.
[12] H. Shi, X. X. Mao, X. X. Chen, Z. H. Wang, K. M. Wang, X. L. Zhu, Biosens. Bioelectron. **2017**, 91, 136.
[13] A. Nikfarjam, A. H. Rezayan, G. Mohammadkhani, J. Mohammadnejad, Plasmonics **2017**, 12, 157.
[14] M. Hansen-Bruhn, L. D. F. Nielsen, K. V. Gothelf, ACS Sens. **2018**, 3, 1706.
[15] X. W. Yan, X. C. Le, H. Q. Zhang, Anal. Chem. **2018**, 90, 9667.
[16] M. V. Kjelstrup, L. D. F. Nielsen, M. Hansen-Bruhn, K. V. Gothelf, Biosensors **2018**, 8.
[17] S. I. Khan, R. R. Chillawar, K. K. Tadi, R. V. Motghare, Curr. Anal. Chem. **2018**, 14, 474.

Author Contributions

H.L. conceived the study, designed and performed the experiments, analyzed the data and wrote the manuscript. H.L. and J.V.S. fabricated the chip together. J.V.S. and M.H.B. offered critical reading and comments on the manuscript. K.V.G. conceived of and supervised the study, and co-wrote the manuscript. All authors reviewed the manuscript and provided comments.
